# From Division to Death: Metabolomic Analysis of *Nicotiana tabacum* BY-2 Cells Reveals the Complexity of Life in Batch Culture

**DOI:** 10.3390/plants13233426

**Published:** 2024-12-06

**Authors:** Roman K. Puzanskiy, Anastasia A. Kirpichnikova, Ekaterina M. Bogdanova, Ilya A. Prokopiev, Alexey L. Shavarda, Daria A. Romanyuk, Sergey A. Vanisov, Vladislav V. Yemelyanov, Maria F. Shishova

**Affiliations:** 1 Laboratory of Analytical Phytochemistry, Komarov Botanical Institute of the Russian Academy of Sciences, 197022 St. Petersburg, Russia; bogdanova.ekaterina15@gmail.com (E.M.B.); prokopiev@binran.ru (I.A.P.); shavarda@binran.ru (A.L.S.); 2 Faculty of Biology, St. Petersburg State University, 199034 St. Petersburg, Russia; nastin1972@mail.ru (A.A.K.); st087848@student.spbu.ru (S.A.V.); bootika@mail.ru (V.V.Y.); 3 Center for Molecular and Cell Technologies, St. Petersburg State University, 199034 St. Petersburg, Russia; 4 Laboratory of Genetics of Plant-Microbe Interactions, All-Russia Research Institute for Agricultural Microbiology, 196608 St. Petersburg, Russia; d.romanyuk@arriam.ru

**Keywords:** BY-2, *Nicotiana tabacum*, metabolomics, plant cell culture, elongation growth, metabolite mapping, batch culture, plant senescence

## Abstract

Tobacco BY-2 cell culture is one of the most widely used models in plant biology. The main advantage of BY-2 suspension cultures is the synchronization of cell development and the appearance of polar elongation. In batch culture, BY-2 cells passed through the lag, proliferation, elongation, and stationary phases. During this process, the composition of the growth medium changed dramatically. Sucrose was rapidly eliminated; hexose first accumulated and then depleted. The medium’s pH initially decreased and then rose with aging. As a result of the crosstalk between the internal and external stimuli, cells pass through complicated systemic rearrangements, which cause metabolomic alterations. The early stages were characterized by high levels of amino acids and sterols, which could be interpreted as the result of synthetic activity. The most intense rearrangements occurred between the proliferation and active elongation stages, including repression of amino acid accumulation and up-regulation of sugar metabolism. Later stages were distinguished by higher levels of secondary metabolites, which may be a non-specific response to deteriorating conditions. Senescence was followed by some increase in fatty acids and sterols as well as amino acids, and probably led to self-destructive processes. A correlation analysis revealed relationships between metabolites’ covariation, their biochemical ratio, and the growth phase.

## 1. Introduction

Cell cultures originated from higher plants have been in use for more than half a century. Such cultivation became possible after phytohormones, as important growth regulators, were discovered [1]. The employment of these compounds allowed undifferentiated actively proliferating cells to be obtained, regardless of their initial specialization in the mother plant. In the early 1960s, Toshio Murashige and Folke Skoog developed the composition of a culture medium. Its various modifications are widely used nowadays [2]. Initially, callus cultures, representing conglomerates of undifferentiated cells, were derived [3]. Cell suspensions were obtained by transferring callus to a liquid medium with agitation. [4]. These have a number of important advantages: greater uniformity, a higher growth rate, and ease of scaling. Along with the possibility of maintaining cultures under controlled aseptic conditions, they allow for highly reproducible results. In this regard, suspension cell cultures have become the models of plant biology [4,5,6,7]. The successes of recent years in the design of cell lines originated from different plant species have found wide application in biotechnology as producers of biologically active secondary compounds [8,9] and heterologous proteins [10,11]. Moreover, there is a potential for using plant cells in the food industry as producers of raw materials for food production [12,13], as well as for cryopreservation and vegetative reproduction of plants [14,15].

Arabidopsis cell cultures [8] and tobacco [16,17] have acquired the greatest importance as model objects. Among tobacco cell lines, BY-2 is the most popular. The history of using this line goes back more than 50 years [18]. BY-2 has exceptional advantages—synchronization of the cell cycle under suspension culture conditions and preservation of polar elongation growth [19,20,21]. The development of BY-2 cell cultures can be divided into four stages. During the lag phase, the cells adapt to the new environment and prepare for division. In the proliferation phase, cells undergo numerous divisions without increasing in volume. After that, the cells grow by elongation, increasing in cell length and volume. After growth completion, cells enter a stationary phase finishing in death [20].

In the process of culture development, cells undergo systemic morphophysiological changes that are associated with corresponding transcriptional, proteomic, and metabolomic rearrangements. It was found that the activity of aerobic respiration of heterotrophic tobacco cells reaches a maximum during the growth period [22], which corresponds to a greater number of mitochondria and peroxisomes [23]. The shape of mitochondria also changes: after subculturing, they acquire a spherical shape, and as they age, they become elliptical. The shape of plastids changes differently. After passaging, plastids become strongly elongated and then fragment, becoming elliptical [24]. Even though BY-2 cells have lost their ability to photosynthesize, plastids continue to play an important physiological role. One of the functions of plastids is the deposition of starch. In the stationary phase, a greater number of starch grains is observed in plastids [23].

The results of transcriptomic analysis of BY-2 cells confirm the large-scale metabolic differences between the lag, log, and stationary phases. The lag and proliferation phases were characterized by high expression of genes associated with DNA translation and replication. Genes associated with lipid synthesis showed expression patterns with maxima both at the beginning of development and in the stationary phase [25]. Cultures of different ages also differ in the accumulation of metabolites. Thus, the key trait of growing VBI-0 cultures compared with senescent ones is a greater accumulation of amino acids and phosphates of sugars and fewer carboxylates and complex sugars [26]. Nitrogen metabolism also changes with age. During BY-2 proliferation, the content of amines in the cells—spermine and spermidine—increases, and putrescine decreased [27]. In addition, after subculturing, a decrease in pH is observed, which is probably the result of organic acid secretion [28,29].

BY-2 cells have lost their ability to photosynthesize; as a result, they are obligately heterotrophic and are maintained in the dark using sucrose as a substrate [16]. Sucrose metabolism begins with cleavage, which is carried out by two types of enzymes: invertases and sucrose synthases. The contribution of these enzymes may vary depending on external and internal factors. Moreover, the cleavage of sucrose can be carried out both in intracellular compartments in the cytosol and vacuole and by extracellular enzymes. The latter leads to the accumulation of hexoses in the medium, which can be absorbed [30]. Sucrose hydrolysis products can be catabolized through glycolysis, the tricarboxylic acid (TCA) cycle, and the pentose phosphate pathway (PPP), directed to the synthesis of amino acids or lipids, or deposited in the form of starch. Obviously, during the culture growth, the substrate is exhausted and starvation develops. The first consequence of starvation consists in physiological activity slowdown [31,32]. In addition, the mobilization of reserves is observed during cell starvation [31,33,34]. High proteolytic activity occurs since amino acids can also be a source of carbon during starvation. This corresponds to an increase in the expression level of enzyme genes associated with amino acid catabolism [35], especially with branched amino acid catabolism [36]. Amino acid catabolism entails a restructuring of nitrogen metabolism [37]. However, starvation of heterotrophic cultures can only last for a very limited period of time. The depletion of nutrients and other environmental changes give rise to a complex of physiological processes that are commonly attributed to senescence, ending in the death of the culture.

The assimilation of the substrate and the metabolic response to its deficiency are primarily associated with central metabolism. A set of primary metabolites, represented mostly by small molecules such as C_2_–C_7_ carboxylic acids, amino acids, monosaccharides, fatty acids, etc., represents a specific metabolic profile characterizing the state of a biological object. A sensitive method for metabolic profiling is gas chromatography coupled with mass spectrometry (GC-MS), which is widely employed for systemic metabolomics analysis [38]. In this work, the metabolites of BY-2 suspension cultures of different ages were profiled. Cells were sampled during the lag phase (the period between inoculation to fresh medium and proliferation), the proliferation phase (the time of active cell division), the expansion growth phase (when the cell elongates after the divisions), and the stationary phase (the period between the end of elongation and death), including the “death” phase (when the number of dead cells rapidly increases). The metabolomic data were compared with data on cell growth, biomass, and substrate uptake.

## 2. Results

### 2.1. Growth and Sucrose Uptake

**Figure 1:**
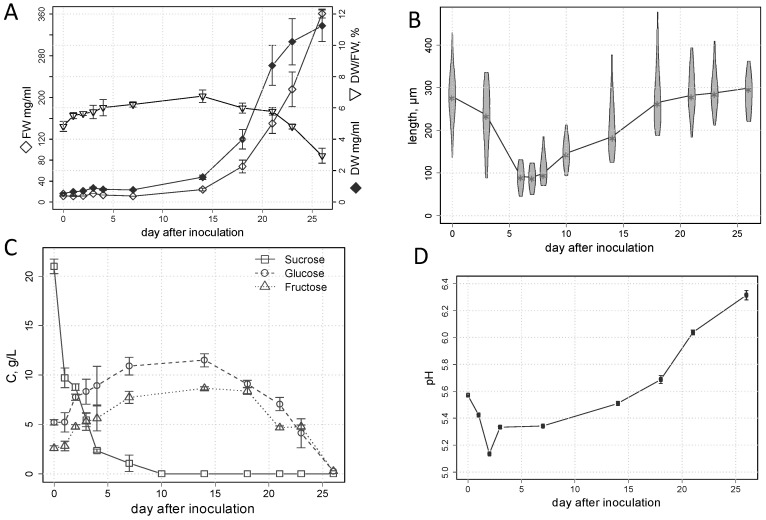
Parameters of the growth of the *Nicotiana tabacum* BY-2 suspension batch culture: Biomass (fresh weight (FW), dry weight (DW), accumulation and its ratio) (**A**); violin plots of cell length distribution changes (**B**); sugars concentration in the medium (**C**); and pH of the medium (**D**).

The BY-2 cultures demonstrated a logarithmic increase in the density of fresh biomass, with a maximum of about 350 mg/mL by the end of culture growth (Figure 1A, Table S3). The dynamics of the dry biomass density were described by an S-shaped curve with a maximum of about 11 mg/mL. The proportion of dry biomass increased to 7% in the first two weeks, then decreased to 3% at the death phase. In the first week, there was almost no increase in fresh biomass, but dry biomass almost doubled. Between the second and third days, the cells began to proliferate, and their length began to decrease (Figure 1B). It should be noted that in the first two days, the concentration of sucrose in the incubation medium decreased by half, and at the 7th day, less than 10% of its initial concentration remained (Figure 1C). After the first day, there was an increase in the levels of fructose and glucose in the medium. At the same time, the pH decreased to 5.1 in the first two days (Figure 1D), but then the pH level began to rise, reaching 6.3 at the death phase. At the 6–7th day, proliferation was completed and the cells began to grow by elongation (Figure 1B). The elongation growth consisted of a sharp increase in the cell length, and the growth rate of fresh biomass. After 18 days, the cells reached their maximum length and the elongation ended. At the 10th day, the sucrose in the medium was exhausted, but hexoses were present (Figure 1C). The hexose content began to decrease after about two weeks and dropped to zero by the beginning of culture death.

### 2.2. Metabolomics: General View

**Figure 2:**
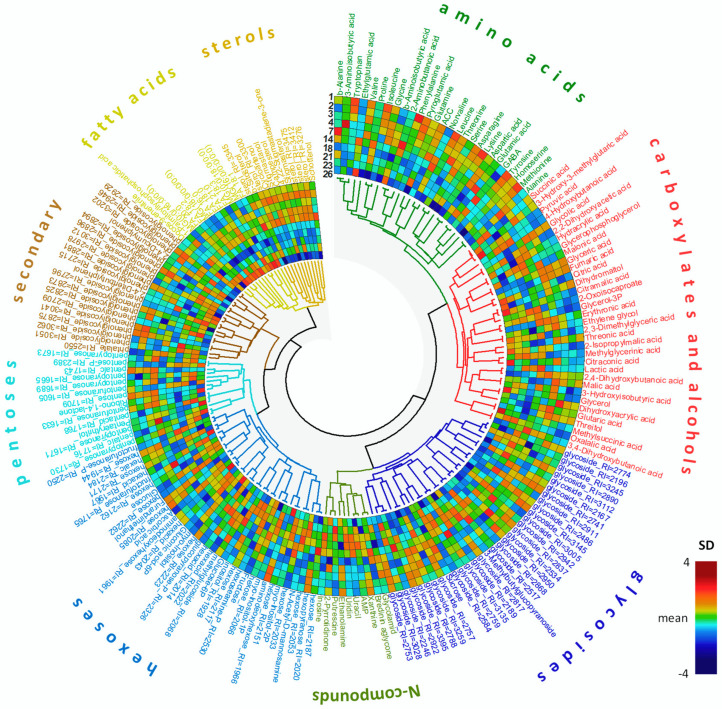
Heat map of the standardized mean normalized metabolite content in BY-2 suspension cultures. Metabolites were divided into groups by chemical class, metabolites within groups and the groups themselves were clustered by Pearson distance and Ward’s method.

As a result of GC-MS analysis, metabolite profiles were obtained, including about 360 compounds. More than a hundred (110) were identified. A chemical class was annotated for another 90 (Figure 2). Sugars and their derivatives (about 90 in total), including pentoses, hexoses, sugar alcohols, sugar acids, phosphates, disaccharides, and various glycosides, were the most numerous in the metabolite profiles obtained. The profiles also included 27 amino acids and about three dozen carboxylic acids, with intermediates of energy metabolism among them. About a dozen free fatty acids and their derivatives and phytosterols were annotated.

**Figure 3:**
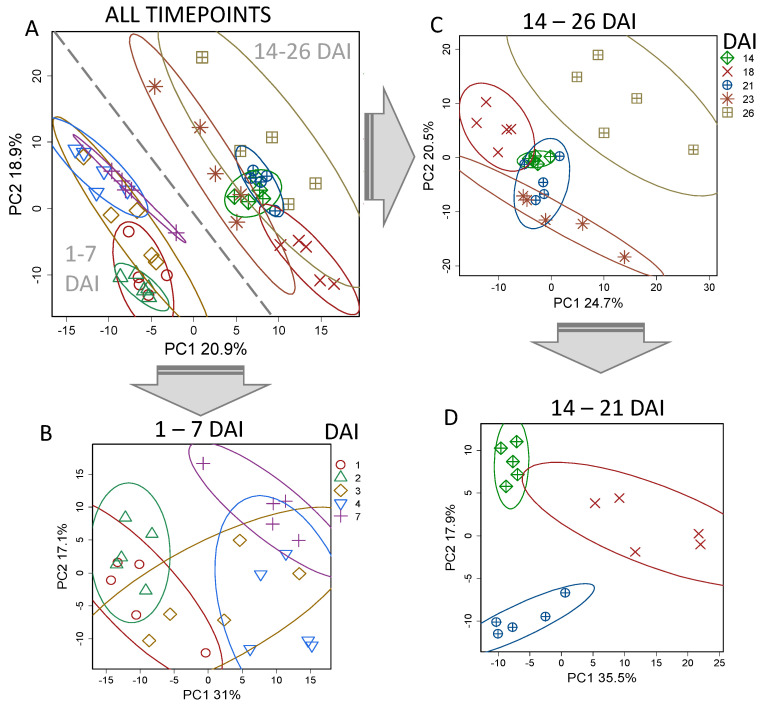
Metabolomic dynamics during development of BY-2 suspension cultures. PCA score plots for metabolite profiles from all tested time points (**A**), the lag and proliferation phases (**B**), period after the division stopped (**C**), and expansion and transition to the stationary phase (**D**). Ellipses are 90% confidence intervals. DAI—day after inoculation.

A PCA (principal component analysis) revealed that the samples were grouped according to the age of the culture (Figure 3). Two separate clusters were formed by cultures in the periods 1–7 days (lag phase, proliferation, and initiation of elongation) and 14–26 days (active elongation growth, stationary phase, and cell death). A further examination of these groups showed that sub-groups according to age were determined within them. For instance, the cultures at the lag phase (1–2 days) differed from the proliferating ones (4–7 days), while three-day cells had an intermediate position. Cultures, starting at two weeks and ending at 23 days, formed close sub-groups. While the profile on day 26, in dying cells, was different.

### 2.3. Changes in the Development Process

#### 2.3.1. Differences in the Lag Phase and Proliferation

**Figure 4:**
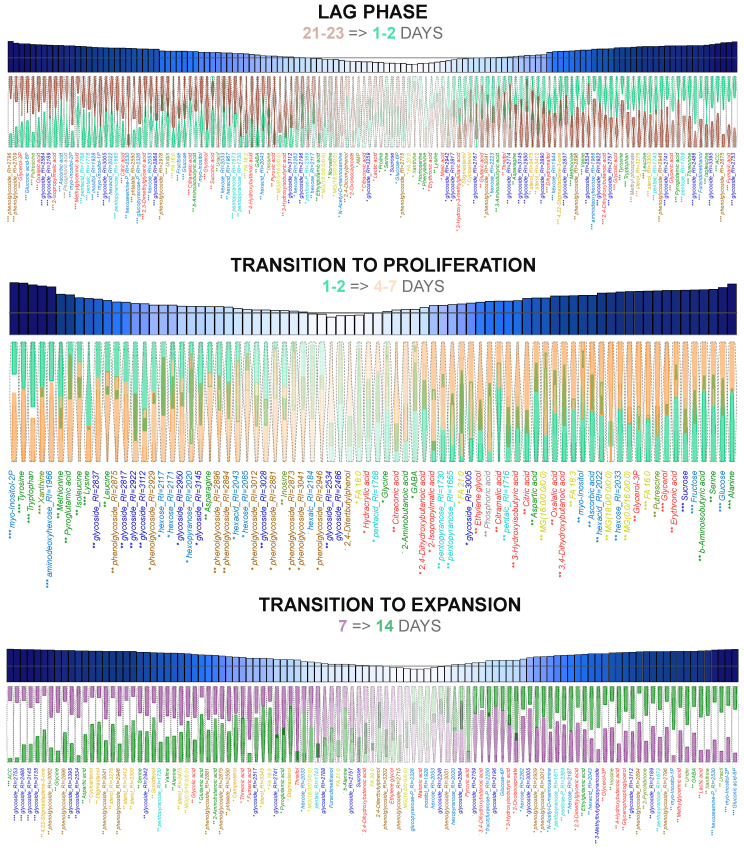
Alterations in metabolite accumulation at early stages of culture development. Violin plots of normalized metabolite content. Colors represents days after inoculation that are mentioned in each plot. Bar plots above are VIPs: gray line marks VIP = 1; stars mark adjusted *p*-values from MMW test for lag phase and transition to proliferation and from *t*-test for transition to expansion (*** for *p* < 0.001, ** for *p* < 0.01, * for *p* < 0.05, ˙ for *p* < 0.1). Dark blue bars correspond to higher VIP values. Compound names colored same as in Figure 2.

**Figure 5:**
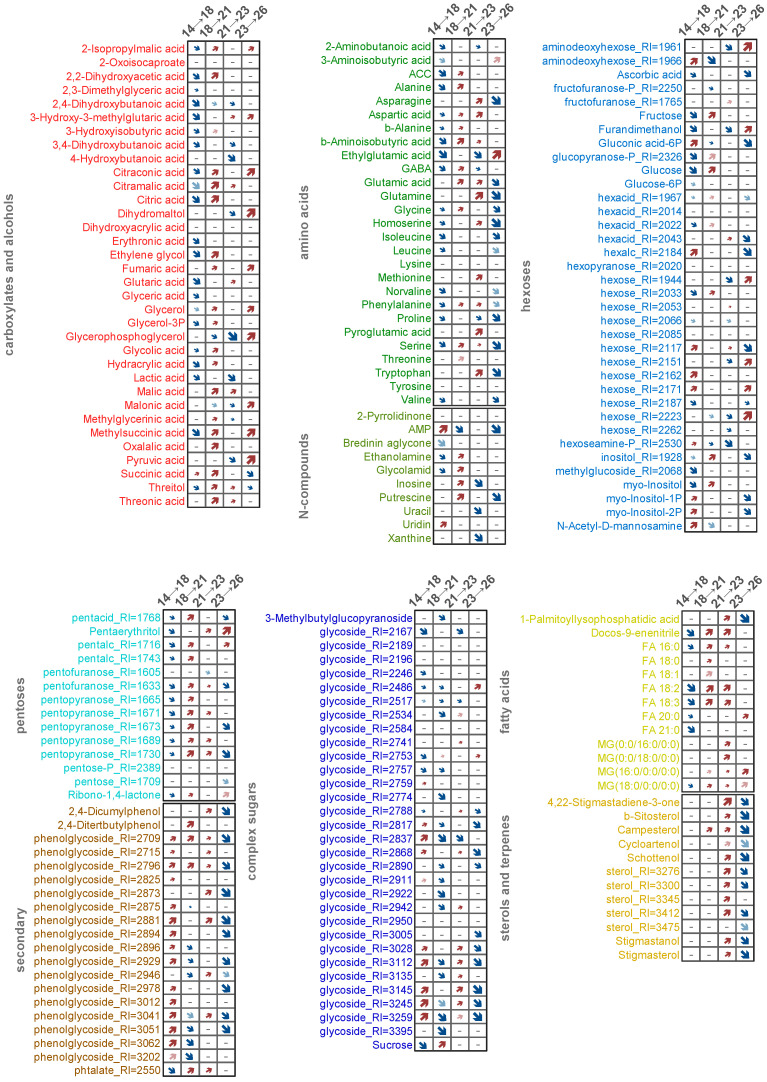
Alterations in metabolite accumulation at later stages of culture development. Heat maps of loadings (p) of predictive component from OPLS-DA models for sequential pairwise comparison: red upward arrows refer to positive p, which correspond to level increasing. Blue downward arrows represent negative p, which correspond to level decreasing. Size of arrows represent strength of alterations. Saturated colors correspond to VIP > 1, pale colors to 0.9 < VIP < 1.

At the next stage, the dynamics of the cultures’ metabolite profiles were analyzed in detail with OPLS-DA (orthogonal partial least squares—discriminant analysis). The result of the selection of differentially accumulated metabolites is shown in Figure 4 and Figure 5. The parameters of the obtained models are shown in Supplementary Table S2. First, we determined what changes occur in the period after passaging (during the lag phase). Thus, a comparison of 21–23-day cultures (the time point of subculturing) and newly transferred 1–2-day cultures was performed. This showed that 39% of the variance was associated with the predictive component. As shown in Figure 4, newly transferred cells accumulated sterols during the lag phase, including stigmasterol, which is a key metabolite for plants. The increase in level was typical for many amino acids, including aromatic ones: phenylalanine, tyrosine, and tryptophan. On the contrary, several amino acids were characterized by a slight decrease in their level, including GABA (γ-aminobutyric acid) and proline. The content of intermediates of energy metabolism changed. The pool of intermediates from the second part of the TCA cycle, fumarate and malate, increased, while the initial intermediates, citrate, succinate, and pyruvate, showed a reduction. The level of glycolate, a metabolite associated with the glyoxylate cycle, was also elevated. There were serious changes in carbohydrate metabolism. Despite the active metabolism of sucrose, its content in cells showed a downward trend, as did the accumulation of fructose, glucose, and its phosphorylated forms. Also, there was a noticeable redistribution in the accumulation of glycosides, and a decrease in the accumulation of FFAs (free fatty acids). A sharp increase in the level of ACC (1-Aminocyclopropanecarboxylic acid), a precursor of ethylene, deserves special attention.

**Figure 6:**
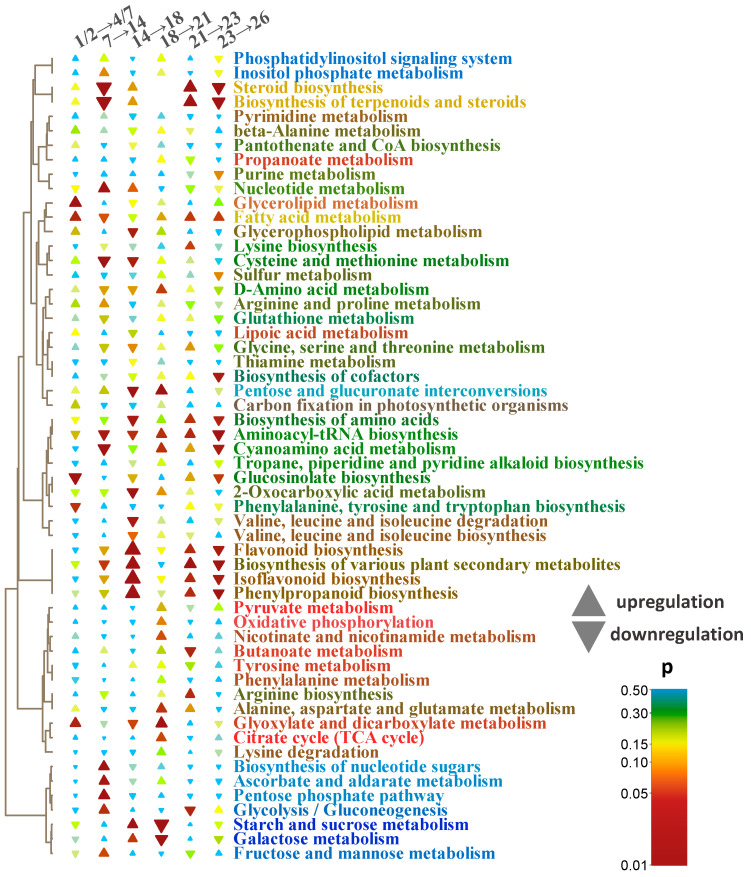
Metabolite set enrichment analysis based on OPLS-DA loadings. The size of the triangles represents the absolute NES (normalized enrichment score) value, which reflects the strength of the effect. Upward triangles refer to positive NES (generally, up regulated) and vice versa. Pathways clustered by a number of common metabolites in the profiles. The colors of the pathways are mixes of the compound colors as shown in Figure 2.

A subsequent analysis of the metabolomic changes during the proliferation and initiation of growth was carried out based on a comparison of 1–2-day with 4–7-day cultures. The time points were selected based on the grouping of samples during the PCA (Figure 3). In the resulting model, 31% of the variance was associated with the predictive component. As shown in Figure 4, cultures in the first 2 days are characterized by a relatively high content of amino acids with hydrophobic side chains, including the aromatic tyrosine and tryptophan, aliphatic ones such as leucine and isoleucine, and sulfur-containing methionine. Accumulation of pyroglutamate and asparagine was also noted. Higher levels of aromatic amino acids were consistent with bigger pools of aromatic glycosides. However, a large number of other glycosides were also elevated in the first two days. Further on, at 4–7 days, the content of alanine, serine, aspartate, and glycine increased. Also at this stage, there was a tendency to increase the levels of non-standard C_4_ amino acids: GABA, 2-aminobutanoic acid, and β-aminoisobutyrate. Among other nitrogen-containing compounds, a decrease in the content of inosine and xanthine and, conversely, an increase in putrescine should be highlighted. A striking difference between the 4–7-day and 1–2-day cultures is the higher accumulation of carboxylates. Herewith, the only intermediate of the TCA cycle was present among them—citrate. Another distinctive feature of the 4–7-day cultures is the increased content of FFAs and acylglycerols, as well as glycerol and glycerol-3P. Interestingly, the levels of sucrose, fructose, and glucose showed an increase and reached a maximum at this stage. There were changes in the composition of glycosides. MSEA revealed (Figure 6) that between 1–2 and 4–7 days, fatty acid and lipid metabolism is activated while the metabolism of aromatic amino acids and glucosinolates is suppressed.

#### 2.3.2. Cell Elongation Stage

As shown by the dimension reduction, the most pronounced changes occurred during the active elongation growth between 7 and 14 days (Figure 3). It is noteworthy that the culture metabolic profiles at the elongation phase (14 days) formed a very dense group, which indicated their high homogeneity during this period. A total of 42% of the variance was associated with the predictive component of the OPLS-DA model. This stage was characterized by a decrease in the content of numerous sterols (Figure 4), acylglycerols, and FFAs. Multidirectional trends were identified for carboxylates. Particularly, the content of fumarate and glycolate was characterized by a decrease, while the lactate and pyruvate levels increased. A lower accumulation was noted for several standard amino acids. Among non-proteinogenic amino acids, GABA demonstrated elevation, in contrast to ACC and β-alanine. There was an increase in the content of nitrogenous bases and nucleosides. Thus, MSEA has shown (Figure 6) that intensively elongating cells have a reduction in the metabolism of sterols and fatty acids. Weakening also occurred in the sulphur-containing and cyanoamino acid exchange pathways. At the same time, a decrease in the activity of the protein synthesis pathway was also observed. In addition, the synthesis of secondary compounds was suppressed but the levels of ascorbate, pentose, and nucleotide metabolism increased.

#### 2.3.3. Elongation Growth Completion and Transition to the Stationary Phase

The next step was the comparison of the metabolic profiles at later stages of growth: completion of the cell elongation stage (14 day) and transition to the stationary phase (18 day). A total of 43% of the variance was associated with the predictive component of the OPLS-DA model. The end of elongation growth was characterized by a radical drop in the content of carboxylates, amino acids, pentoses, and FFAs (Figure 5). The exception was succinate, whose level increased. There were multidirectional trends among hexoses: decreases in glucose, fructose, and myo-inositol occurred. At the same time, the pools of 6-phosphogluconate and inositol phosphates were also reduced. The most intense accumulation was noted for aromatic glycosides. Other glycosides showed multidirectional changes, among which a further decrease in the sucrose level seems important. Nevertheless, no alterations of sterols and acylglycerols were determined. It is noteworthy that the AMP level increased. MSEA (Figure 5) demonstrates that against the background of the repression of various pathways, the synthesis of secondary compounds and glycoside metabolism are activated. The predictive component of the OPLS-DA model for comparing 18- and 21-day cultures was associated with 38% variance. The completion of elongation growth and inhibition of biomass growth were marked by the accumulation of carboxylates, including intermediates of energy cycles (Figure 5): malate, fumarate, citrate, succinate. Along with them, the levels of a number of amino acids and amines increased. There was also an increase in the accumulation of sugars, including sucrose, glucose, and fructose, as well as numerous pentoses. However, a decrease in the pools of a large number of glycosides was noted. Acylglycerols and fatty acids also accumulated to a greater extent at the 21st day. The further aging of cultures caused relatively little change in metabolite profiles between the 21st and 23rd days. In the OPLS-DA model, only 22% of the variance was associated with the predictive component. A striking feature of this transition was an increase in the content of lipophilic compounds, including major sterols and fatty acid derivatives (Figure 5 and Figure 6). Glycosides and pentoses also showed a general tendency to accumulate. Accumulation of a number of standard amino acids was noted, while the content of GABA, proline, and nitrogenous bases decreased.

#### 2.3.4. “Death” Stage of Cell Culture

The difference between the 23- and 26-day-old cultures, when the cultures began to die, was slightly more pronounced. In the PLSDA model, 29% of the variance was associated with the predictive component. In general, the dying cultures were characterized by a reduction in the levels of a large number of metabolites (Figure 5). This reflected the repression of sterol metabolism, and the synthesis of secondary compounds and amino acids (Figure 6). Some accumulation of acylglycerols, FFAs, and glycerol was observed. In addition, a number of carboxylates increased their content, among which fumarate can be distinguished. It should be noted that the levels of some sugars were relatively high during this period.

### 2.4. The Relationship Between the Metabolite Pools Volumes

**Figure 7:**
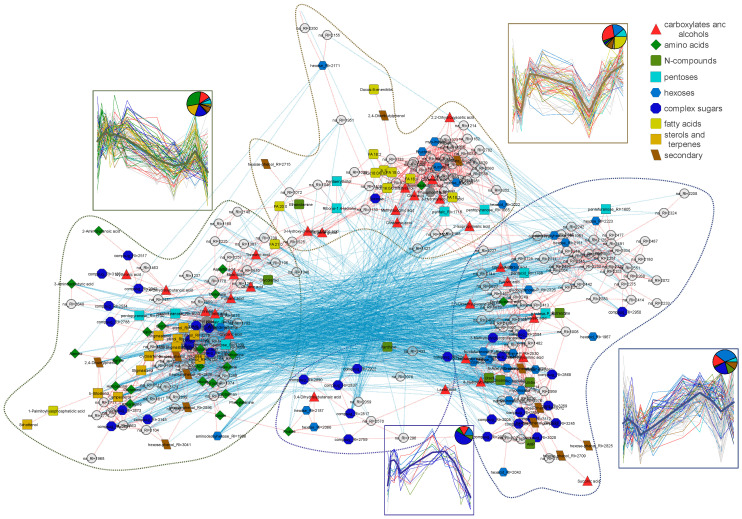
Metabolite mapping by the correlations of the content. Mapping of metabolites by strong (r > 0.85) correlations of their mean levels at different time points. Nodes correspond to the metabolites, the edges correspond to a strong correlation: red—positive, blue—negative. Positive correlations contract nodes. Dotted lines are boundaries of clusters revealed by k-means in coordinates of nodes. In boxes, patterns of dynamics for metabolites: bold line is a median, pies represent proportion of classes of metabolites in the cluster.

**Figure 8:**
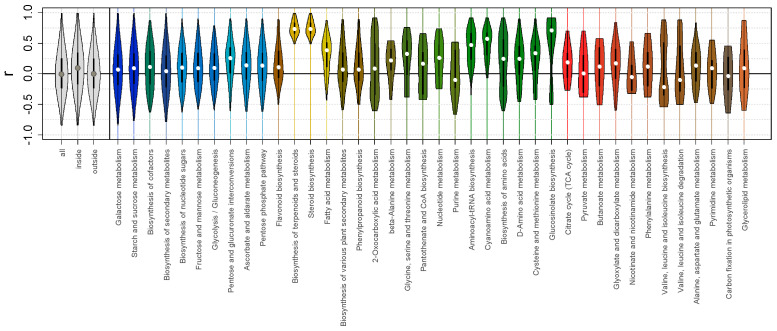
Relation between biochemical pathways and metabolite level variations. Violin plots of Spearman’s correlations (*r*) between all pairs of metabolites (all), inside pathways (inside), outside pathways (outside), and *r* calculated for metabolites of the same KEGG pathways. The colors of the pathways are mixes of the compound colors, as shown in Figure 2.

To identify the relationships between the metabolite pools in the context of the dynamics of physiological status in the process of the culture cells’ development, we mapped the metabolites by correlation of the average normalized values of their concentrations. A graph was constructed where the metabolites correspond to nodes (Figure 7), which are connected by edges corresponding to a strong correlation. They pull the nodes together if the correlation is positive. The metabolites were divided into four clusters by the k-means method. The dynamics patterns for each cluster were illustrated by a broken median change. The first larger cluster (no. 1) combined metabolites with the highest accumulation in young cultures. There were amino acids, sterols, and some intermediates of energy metabolism. Another large cluster (no. 3) combined metabolites with the opposite trend—accumulation over time. It was dominated by sugars, some carboxylates, and nitrogen-containing compounds were present. The metabolites of cluster no. 2 had high levels at the start of culture development and the elongation phase, as well as at the end of development. These included most of the FFAs and carboxylates. Compounds with a similar chemical nature and related metabolically are often located close to each other in a correlation network (Figure 7). This may indicate the effect of metabolic connections on the pattern of changes in the metabolome. To test this hypothesis, the distribution of the values of correlations of all metabolites among themselves was considered (Figure 8, “all”). The values were distributed between −1 and +1 with a median of about 0. Next, the correlation values were determined for pairs of metabolites belonging to the same group identified by KEGG (“inside”). The distribution of the obtained correlation coefficients was shifted in a positive direction, whereas the correlation values for pairs of metabolites that do not have common groups (“outside”) did not show such a trend. Metabolites in different pathways showed diverse correlation similarity in the patterns of dynamics. Higher correlations were observed in the metabolic pathways of fatty acids and sterols, and lower correlations were observed in the metabolism of pyruvate, sugars, and secondary compounds. Among the amino acid exchange pathways, those related to pyruvate and TCA and containing carboxylates as a result showed less connectivity. In contrast, the amino acid exchange groups, those that were more homogeneous and contained fewer carboxylates, showed higher correlations.

**Figure 9:**
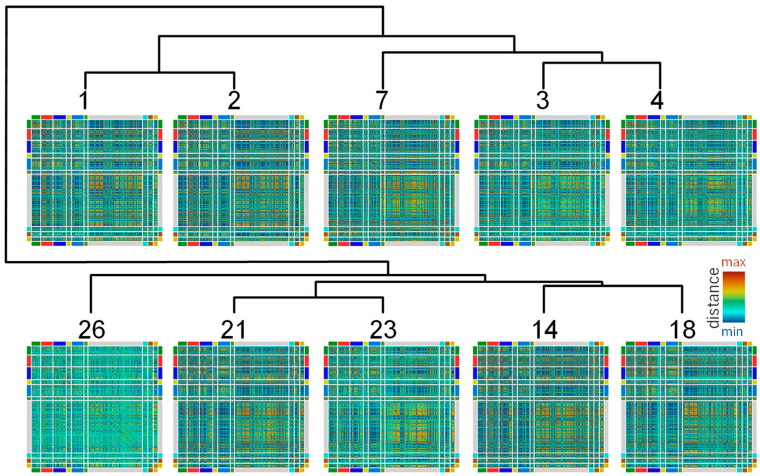
Comparative analysis of patterns in individual variability of cultures. The similarity of metabolite variability was compared by Canberra distance in the space of their normalized standardized content in independent cultures (heat maps). Colored lines at the sides of heat maps mark biochemical classes of compounds, as shown at Figure 2. These pair distances were used as a space for clustering time points by Canberra distance (dendrogram). Numbers—days after inoculation.

Then, the random variability in the metabolite content in cultures of different ages was analyzed. This may be limited by the metabolic links of metabolites. Therefore, its study can shed light on the structure and regulation of metabolic processes at various cell developmental stages and culture conditions. The similarities of the dynamics of the metabolites were estimated by the Canberra distance in the space of their content in every culture (biological replications) at each time point. Next, the time points were clustered according to the similarity of distances for pairs of metabolites (Figure 9). It can be seen that clustering is in good agreement with the development of cells. As in the case of PCA, the profiles are divided into two large groups: the beginning of development on the one hand, and further differentiation on the other. The employed method of the clustering clearly revealed separation of each period. The first one included the lag phase (1–3 days) and proliferation/initiation of elongation growth (4–7 days). The second one combined elongation growth (14–18 days) and aging (14–26 days) and continued with cell death (26th day).

## 3. Discussion

### 3.1. Cell Culture Is a Dynamically Changing System

Like other cell cultures, tobacco BY-2 suspension culture goes through the stages of proliferation, then transits into a stationary phase, after which it dies. The exclusive feature of BY-2 is the elongation growth that occurs after proliferation has completed. This brings the processes of cell development in the culture closer to those in the whole plant. The physiological and biochemical trails of culture cells are in accordance with their development status and alterations in the culture medium. The latter is determined by the exhaustion of the medium’s resources, an increase in density and, as a result, in a gas regime, etc. [39,40]. Cells are also known to secrete various metabolites and proteins into the medium. Thus, the culture medium is considered to be a complex active biochemical system exhibiting traits of the extracellular compartment [41]. The regulation of the process of development of the culture cells seems to be much more complex. And if metabolic changes in microorganisms are caused by environmental factors that trigger adaptation processes, then it is assumed that development programs may be preserved, at least partially, in cells originating from higher plants. These programs are guided by various endogenous and exogenous stimuli, including hormonal ones. The most important hormones affecting the state of BY-2 cells are auxins and cytokinins [42,43,44,45]. BY-2 cells are capable of synthesizing cytokinins; in addition, auxin or its analogs are added to the medium [16]. A shift in hormone levels during culture growth is thought to be one of the drivers of the physiological changes that occur in cells [17,42]. Analysis of 2,4-D (2,4-dichlorophenoxyacetic acid, the synthetic analogue of auxin) content in media showed its decrease in the medium to almost zero by the sixth day of development [28].

The metabolome is one of the integral parameters that reflects the state of the organism [46,47,48]. According to our data, the metabolite profiles of tobacco suspension cells differ at different stages of cultures growth (Figure 2 and Figure 3). It is important that links between pools of different metabolites are also altered (Figure 9). Similarly, the metabolite profile changes triggered by aging were earlier determined in plants over longitude experiments [49,50,51,52]. Moreover, those changes were specialized according to the developmental and/or physiological stage of the studied organ or tissue [53,54,55]. The profile of metabolites changes significantly during cellular differentiation, including elongation growth. For example, during the growth of flax fibers [56].

### 3.2. An Important Role in the Consumption of Sucrose Is Played by Its Extracellular Cleavage

Sucrose is the main source of carbon in heterotrophic cultures and plant organs. It is the main and universal transport form of carbon in higher plants [57,58]; this explains the possibility of its metabolization by cell cultures [59,60,61]. Sucrose uptake is provided by several transport systems, including SUTs (sucrose transporters), MSTs (monosaccharide transporters), and SWEET (sugars will eventually be exported transporters) [62,63,64,65]. Along with this, the breakdown of sucrose by extracellular invertases may occur, as well as the subsequent absorption of the end products. Accumulation of glucose and fructose in the medium of some cultures, including BY-2, has been observed to be accompanied by a decrease in the sucrose pool, pointing to the activity of extracellular invertases [40]. In addition, the maximum specific level of sucrose consumption preceded those of glucose and fructose; therefore, sucrose hydrolysis was supposed to occur prior to absorption [66]. BY-2 cells were shown to excrete proteins into the medium, and also acidify it with carboxylic acids, for example, uronic acid, especially during the lag phase of culture development [29,67]. All these are in agreement with our data. Three stages in the consumption of sucrose were revealed. At the first day, there was a drop in the level of sucrose, but its hydrolysis products did not accumulate in the medium. This indicated, apparently, a high absorption rate of the original substrate (Figure 1A). Then, sucrose hydrolysis took place outside the cells with the accumulation of hexoses. Both periods were accompanied by a decrease in pH, which could contribute to the hydrolysis of sucrose. Further on, the hexose was absorbed for a longer time. A similar pattern of sugar consumption was observed in previous studies [39,40,68,69,70]. Hence, a change in substrate nature occurred during the process of culture growth and thus affected the metabolism. Herewith, the levels of sucrose, fructose, and glucose in cells began to grow strongly at the 3rd day and reached a maximum at the 4th day, when about 90% of the sucrose had already been absorbed (Figure 1C).

### 3.3. Proliferation Requires Preparation, Accumulation of ‘Building Blocks’, and Changes in the Krebs Cycle

After passaging, the cells do not begin dividing immediately. A lag phase for BY-2 cells lasted about two days, under the conditions of this experiment. For model microorganisms, the lag phase is known to be a complex dynamic period of systemic preparation for division. The profiling of BY-2-cell metabolites showed that the lag phase (1–2 DAI) was well separated from the division phase, finishing with the transition to elongation (4–7 DAI) (Figure 3). Previously, the absence of an increase in the DNA level for BY-2 cells was shown for the first two days of cultivation, but this was followed by a dramatic increase between the 2nd and 4th days [28]. In our experiments, significant changes in the metabolome occurred during the transition to proliferation. Similarly, it has been shown that a rapid large-scale metabolic rearrangement due to the preparation for division involves the following processes: the repair of damage accumulated in the late stages of the previous culture cycle as a result of stress; the activation of the synthetic apparatus; the accumulation of primary metabolites, etc. [71]. Our data indicated that from the first days after subculturing, the BY-2 suspension cultures increased dry biomass as a result of rapid substrate metabolism (Figure 1A,C). A similar pattern has already been observed in sucrose-supplied media [72]. This reflects that sucrose is the main specialized form of carbon transport, and the cells of higher plants constitutively express the components of its absorption and metabolism. However, this could also reflect a prolonged cell cultivation in a medium with sucrose, which is supposed to promote the selection of cells with sucrose utilization mechanisms that are expressed constantly or rapidly induced after transferring to a new medium. The substrate uptake mechanism can be induced by itself, as is usually the case with microorganisms [73,74]. In addition, the carbohydrate metabolism is controlled by crosstalk between carbohydrates and hormonal signaling. Hormones have been shown to affect both sucrose transport and its metabolism [75,76]. The assumption is that the trophic behavior of plant cells in suspension mirrored a number of control mechanism that have been developed at the tissue and whole-organism levels. For example, in angiosperms, dividing plant cells are organized into meristems, known as the acceptor zones, supplied with sucrose. Moreover, the tips of asparagus shoots, known for meristematic activity, are distinguished by a high content of sucrose from the underlying zones, in which cells grow by elongation, specialize, and senesce [77], as well as culture cells in the proliferation phase (Figure 4).

The accumulation of dry biomass during the lag phase may be caused by the formation/change of cellular structures that were damaged during the previous cycle of culture development as a result of cell starvation and aging. The first day after passaging is accompanied by a drastic elongation of plastids followed by their fragmentation [24,78]. Mitochondria, during the lag and log phases, become granular from being elliptical and their number increases. These changes are accompanied by active organelle DNA synthesis, which is detected in the first hours after subculturing and precedes the replication of nuclear DNA, lasting about 5–7 days during the lag and log phases [24,79]. Along with this, a synthesis and accumulation of metabolites that will be intensively utilized during proliferation occur. These reserves are needed to maintain a high intensity of metabolic fluxes during division. The genes encoding proteins that are involved in the processes of transcription, translation, lipid metabolism, cytoskeleton reorganization, and endomembrane formation were shown to be expressed more intensively during the lag and log phases in BY-2 cells [25]. Similarly, heterotrophic cells of tomato fruits are characterized by more active synthetic processes during the proliferation period and consist of the accumulation of amino acids, lipids, nucleic acids, etc. [80,81,82]. The maximal accumulation of amino acids, many carboxylates, and short FFAs, are also associated with the growth of microalgae cultures [83]. A high content of amino acids is observed at earlier stages of the development of various plants, for example, peas [84] and soybeans [85]. In addition, the apical meristem of asparagus shoot tips is characterized by high levels of amino acids such as glutamate, cysteine, histidine, and phenylalanine [77]. In our experiment, the BY-2 tobacco cells accumulated free standard amino acids during the lag phase and maintained them at a high level during the proliferation period. Nevertheless, a decrease in the levels of several amino acids accumulated in the lag phase, especially aromatic ones, and methionine was detected, which probably indicated its participation in the synthesis of proteins and other compounds. On the contrary, alanine, serine, and aspartate levels increased precisely at the stage of proliferation. The accumulation of nitrogen metabolism intermediates also changed during the development of cell cultures. The level of putrescine first dropped down during the lag phase and then lifted during division. This finding is consistent with previous observations that the period of active growth of BY-2 cultures is characterized by higher levels of free and water-soluble conjugates of amines, such as spermine and spermidine, and a reduction in putrescine. This is accompanied by changes in the activity of polyamine synthesis enzymes [27].

Glycolysis and the TCA cycle are closely related to the synthesis of amino acids. During the lag phase, the levels of pyruvate, citrate, and succinate decreased, in contrast to the levels of carboxylates of the Krebs cycle end (malate and fumarate) which indicated an alteration in the operating mode. The first assumption is that the incoming flow in the TCA cycle was decreasing. However, activation of the glyoxylate shunt and redirection of TCA cycle intermediates into amino acid synthesis are possible. This is in agreement with an increase in the level of amino acids, primarily derived from pyruvate and oxaloacetate. An additional driver of these changes may be a lack of oxygen, since the glyoxylate shunt cuts off part of the reactions requiring oxidized cofactors. During the transition to division, the levels of citrate and many carboxylates increased, reflecting a requirement for energy. Carboxylate pools play an important role in the regulation and stabilization of the TCA cycle [86,87,88]. The maximal activation of aerobic respiration of heterotrophic tobacco cells during the period of active proliferation was revealed previously [22]. This corresponds to an increase in the number of mitochondria and peroxisomes in culture cells [23].

At the stage of the lag phase of the BY-2 culture, the accumulation of sterols was noted, as well as elevation of the FFA level during the proliferation period (Figure 2). Both processes can be associated with the development of membranes. The need to synthesize these membranes is determined by their previous destruction during the aging process of the cells. It is intensified by the increase in the number of organelles occurring before and during the division [78]. An additional reason for the accumulation of FFAs could be a process preceding elongation growth. Similarly, the accumulation of FFAs and lipids was shown at the early stages of germination, before the rapid growth of the pollen tube [89]. The elevation of the sterol level also precedes division. These metabolites have more complex and prolonged synthesis and play a key role in membrane activity [90,91]. Phytosterols’ importance is associated with maintenance of membrane fluidity, permeability, organization, and regulation of the activity of transmembrane proteins, including H ^+^ ATPase []. Thus, its role in the regulation of cellular metabolism, as well as in the process of elongation growth, is assumed. The intensification of membrane protein activity in the course of both processes may be associated with an increase in the number of rafts. It has been shown that rafts in tobacco cell membranes contain significant amounts of phytosterols, predominantly stigmasterol. They contain sitosterol and other sterols in smaller amounts [92]. Taken together, the phytosterols pool is considered as an indirect indicator of the raft formation and the stigmasterol/β-sitosterol ratio is an important pointer of membrane activity [93,94]. In our experiment, this ratio increased during transition to proliferation and then declined during active elongation (Figure S1).

### 3.4. Cell Cycle Regulation Is Linked with Ethylene

The accumulation of ACC in the latency and proliferation phases of BY-2 cultures revealed in our study deserves special attention. Known as a precursor in ethylene synthesis, it has an independent signaling path [95,96,97]. Plant cell cultures were shown to synthesize significant amounts of ethylene, especially during the proliferation period [98]. It is supposed that ethylene is associated with the regulation of the cell cycle and cell elongation [99] but the data are contradictory in a way. The increase in ACC content during the lag and proliferation stages is consistent with the view that ethylene stimulates the transition of cells to the S-phase and inhibits elongation [98]. Recently, an ACC content decrease has been documented for BY-2 culture during senescence, while the level of ethylene increased [72]. The existence of a crosstalk between ethylene and auxin signaling may also play and important role. In suspension cultures of Ruta graveolens, ethylene synthesis was stimulated by both the addition of 1-NAA and, to an even greater extent, of 2,4-D [100]. However, the ACC content increase in BY-2 cultures was upregulated only by very high concentrations of 2,4-D, while the pattern of ethylene content varied in a more complex way [28]. Since BY-2 tobacco cells do not synthesize auxins, and 2,4-D is added to the culture medium, it can trigger the accumulation of ACC and ethylene synthesis after subculturing. In addition, the level of methionine, a precursor of ACC, also increased at the beginning of culture development (Figure 2 and Figure 4).

### 3.5. Active Elongation Growth Is Accompanied by Activation of Carbohydrate Metabolism and Repression of Synthetic Processes

Elongation growth is one of the most important steps of plant cell differentiation. A rapid longitudinal increase in cellular size is based on osmotic water absorption. Elongation growth is rarely determined during cultivation. BY-2 culture cells are among the few that retain this facility to elongate [20]. After 7 days, the length of the BY-2 cells began the active increase (Figure 1B). This step was associated with radical changes in metabolism, which is in agreement with what has been observed in native plants. Similar processes, for example, occurred during the growth of cotton fibers [56]. According to our study of BY-2 cultures, the period of active elongation growth (7–14 DAI, days after inoculation) and its finalization (18 DAI) were associated with the most intensive metabolic rearrangements (Figure 3).

Sugars, especially sucrose, are the main osmotically active metabolites imported by plant cells and may be accumulated in the central vacuole [101]. In our experiment, cells increased sucrose content during the lag phase and proliferation, which was accompanied by its rapid decrease in the medium. Thus, prior to active elongation a large osmotic sucrose gradient was generated. An important difference between cells growing in a native plant is the continued supply of sucrose due to the influx from donor cells/tissues. On the contrary, in BY-2 culture, elongation growth developed when sucrose was exhausted in the medium (Figure 2 and Figure 3). Thus, the importance of internal sugar reserves is revealed.

Modeling has shown that in tomato pericarp cells, elongation growth in terms of ATP costs is comparable to cell division in culture [80,82]. Energy metabolism is closely related to carbohydrate metabolism. During this period, there are still quite a lot of hexoses in the medium, which can be absorbed and used for various purposes. It was previously established that elongation growth is associated with high activity of enzymes associated primarily with the lower part of glycolysis, whereas the flow through the upper part of glycolysis may decrease [101,102,103]. In our experiment, high levels of phosphorylated hexoses and pyruvate, together with a decrease in the content of sucrose in cells and hexoses in the medium, indicated a high activity of the entire glycolysis pathway. The high content of hexose phosphates with a low ATP/ADP ratio is probably a mechanism for ensuring intensive metabolic flow through glycolysis. The flip side of the combination of high energy costs and carbohydrate catabolism restrictions is the repression of synthetic processes. During elongation growth, the accumulation of amino acids, sterols, and acylglycerols decreased (Figure 2 and Figure 3). It can be assumed that at this step the synthesis of new components stopped and structures and molecular pools accumulated earlier were used. A similarly high level of accumulation of TCA cycle intermediates and amino acids was observed in the initiation phase of cotton fiber elongation. When elongation intensified, their level decreased significantly. At the same time, the accumulation of glucose-6P and hexokinase transcription increased [56]. Interestingly, the accumulation of carboxylates is observed during pollen germination and pollen tube growth [89]. In our experiment, the levels of different carboxylates in tobacco cells decreased directly during the proliferation period and initiation of elongation (4–7 days). However, during the active elongation growth, the pattern of alterations in the carboxylic acid pool changed (Figure 4). In addition, carboxylates are the products of incomplete oxidation of sugars and play a supportive role in maintaining osmotic pressure [101]. Interestingly, both in cotton fibers and BY-2 cells elongation is accompanied by an increase in GABA content. But this increase was not observed in mutant cotton defective in fiber growth [56].

### 3.6. Elongation Growth Completion Is Associated with the Activation of Specialized Metabolism

Over time, the growing conditions of suspension cultures worsen. An exhaustion of nutrients, accumulation of metabolic products, deterioration of the gas regime, alkalinization, etc., commonly occur. The level of ascorbate is considered as a physiological indicator of optimal conditions after subculturing. A low level of this metabolite is detected during the lag phase and proliferation, and then it increases. Similarly, a low level of ascorbate also characterizes apical meristems, both root and shoot [77,104]. Plant organisms are constantly improving their adaptation mechanisms to stress factors. This includes the synthesis of compounds belonging to the group of secondary metabolites of various classes, which might have a wide application. Cell cultures originated from plants are also actively in use as producers of such metabolites. Thus, comprehension of the cross interaction between primary and secondary metabolism during the development of cultures is necessary. One of the frequent responses under stressful conditions is the synthesis of aromatic compounds such as phenylpropanoids [105]. An accumulation of aromatic compounds was observed in senescent cultures, for example, in the case of *Cannabis sativa* L. [106]. Various glycosides, originated from the developed carbohydrate metabolism, are precursors for the synthesis of natural substances. In the metabolic profiles, we detected several dozen compounds which were annotated as glycosides. But exact identification of these is usually complicated due to the huge diversity of representatives and the fact that sugar residues give similar masses in the spectra. According to the mass spectra, we identified those glycosides that potentially have aromatic groups. The BY-2 cell analysis indicated that the completion of elongation growth was marked by a large-scale accumulation of glycosides, including almost all annotated as “phenolic” (Figure 4). It is also important to note the elevation of the aminated sugars level. All this can be interpreted as a non-specific feedback reaction to worsening conditions. However, the list of secondary metabolites synthesized in cell cultures is less diverse than that of native plants. For example, tobacco cells of BY-2 culture did not contain nicotine and nicotinic acid, in comparison to seedlings analyzed with similar methods [107]. This fact leads to the assumption that cells in suspension culture are not under such stressful conditions. Moreover, the nicotine content in tobacco leaves increases with age, and therefore may represent a programmable developmental trait that might be absent in suspension [52].

Counting a close interaction between both forms of metabolism through energy and precursor supply, the intensification of secondary compound synthesis could have an antagonistic relationship with biomass accumulation. This is consistent with the observed reduction in primary metabolites, opposite to the glycoside level (Figure 5). But tobacco cells in this period were characterized by a high content of phosphorylated sugars. Those compounds are known to be precursors in the synthesis of glycosides. However, phosphorylation of sugars with limited catabolism can lead to a metabolic imbalance, associated with a strong decrease in the NTP level. Thus, the observed increase in AMP content can be interpreted as a result of a decrease in nucleotide phosphorylation.

### 3.7. An Alteration in the Composition of the Cell Wall May Be a Driver of Metabolic Changes

The cell wall composition (CW) can be an important sign of carbohydrate metabolism. It has an important contribution, which is about 30%, in the dry mass of BY-2 cells [67]. The process of cell division and differentiation is accompanied by intensive alterations in cell walls. During division, plant cells form a primary CW. Its composition permits regulation of extensibility and further elongation growth. After completion of these developmental stages, the cells synthesize a secondary CW enriched with phenolic compounds. Additional modifications may be formed in the CW as a specialization [108,109]. Hence, analysis of the cell wall composition of BY-2 cells did not reveal any significant changes during the initial period of culture growth (2–6 days), when the majority of the polysaccharide monomers were glucose [67]. In recent studies, it was found that the composition of the CW varies throughout the culture development cycle [28,29]. After the completion of BY-2-cell proliferation, the CW was enriched with cellulose, while the proportion of glucose in the composition of polymers increased [29]. In our study, the glucose level in the metabolite profiles was also detected, but after the completion of elongation, between 18 and 21 days (Figure 5). And the observed accumulation of phenolic glycosides at the end of the cell elongation phase could reflect the formation of secondary CWs. Previously, it was demonstrated that an important factor in the formation of CWs of tobacco BY-2 cells is continuous mechanical action due to constant rotating of suspension cultures, which aimed at preventing cell sedimentation and a possible hypoxic effect. As a result, the pectin shield appears to increase cell protection [72]. A further increase in culture density would intensify mechanical stress, causing changes in the synthesis of pectins and hemicelluloses. In addition, CW carbohydrates are able to absorb water, which may be the reason for an imbalance between fresh and dry biomass (Figure 1A) and the observation of slimy cells in the late stages of development.

### 3.8. Destructive Processes of the Stationary Phase Lead to Secondary Accumulation of Amino Acids, Sterols, and FFAs

The deterioration of cultivation conditions causes growth arrest and transition to a stationary phase. Cell senescence within plant organs and culture development, as well as during starvation, has both common and specific signs. The common ones include a decrease in physiological activity [31] and the induction of destructive processes. For example, during the transition of BY-2 cultures to the stationary phase, the number of mitochondria and peroxisomes in cells decreases [23], and the Golgi apparatus degrades [110]. The progressing proteolysis may lead to some accumulation of free amino acids [111,112]. Catabolism plays an important role in a cell’s energy supply during starvation or stress [37,113,114]. Proteolysis and the linked accumulation of free amino acids during senescence within plant organisms provide remobilization and redistribution of nitrogen [53,115]. The main mobile form of amino groups is nitrogen-rich glutamine [53]. In our study, glutamine, as well as malate, accumulation occurred at the stationary stage of BY-2 culture development. An increase in the content of malate and amino acids during senescence was also demonstrated by suspension cultures of *Arabidopsis thaliana* (L.) Heynh. [35]. Malate accumulation in plant cells is known to be intensive and plays an important role in balancing metabolism [116]. Its pool is supposed to be the final carbon stock during the destruction of cellular components, inhibition of respiration, and inability to export in culture conditions.

At the end of cell culture development, lipid metabolism also undergoes serious changes. A substantial part of the lipid metabolism genes in BY-2 culture cells were activated at the stationary phase [25], and they were supposed to be due to membrane destruction. However, the simultaneous reduction in respiration and the decrease in peroxisome number [23,117] should prevent beta oxidation of FAs and probably lead to their accumulation. The metabolic profiles of BY-2 cells obtained in our experiments showed that at the stationary phase, the level of FFAs increased first, followed by free sterols. The later accumulation of phytosterols was probably associated with the multistep destruction of membrane structures, for example, rafts.

### 3.9. The Death of the Culture Is Accompanied by Catabolism and Weakening of Defense Reactions

Figure 1C shows that the period of cell death in the BY-2 suspension culture corresponded to a drop in the level of hexoses in the medium to a level close to zero. Perhaps carbon starvation becomes the most important driver during this period. It led to a decrease in metabolic activity, cell degradation, and subsequent death [37]. The percentage of dry biomass decreased to a minimum, which indicates the mobilization of reserves (Figure 1A). Furthermore, the levels of amino acids and sterols exhibited a decline and reached a minimum. Nevertheless, the levels of FFAs and acylglycerols remained quite high. This is probably the result of lipid degradation with a lack of beta oxidation. The protective reaction associated with the synthesis of glycosides also faded. The detected accumulation of pyruvate and carboxylates could indicate the activity of the lower part of glycolysis as well as catabolism of amino acids and sterols. Nevertheless, resource mobilization cannot ensure the long-term viability of starving cell cultures originated from angiosperms. This is in stark contrast to microalgae, which can remain viable during long-term limitations of external energy and carbon sources [118]. The assumption is that cells of angiosperm plants metabolize degradation products of lipids and proteins as a carbon source at a much lower extent and are much more sensitive to the external supply of resources.

### 3.10. Coregulated Blocks of Metabolites

Many different tools and approaches exist to assess the relationships between metabolites [119]. The main one is the correlation between their content [120]. Since correlation considers the sum of all metabolic reactions, transport, and regulatory processes, it can be considered as a property of the entire system [119,121]. One way to represent correlations is to construct a graph where the edges reflect the presence of a strong correlation. The analysis of such graphs makes it possible to identify systemic patterns of metabolic changes occurring in biological objects [122]. Our mapping of strong (r > 0.85) correlations of the average level of metabolites at all studied time points is presented in the form of a graph (Figure 7). Biological networks are characterized by a scale-free structure. Such networks are characterized by heterogeneity and the presence of a small number of nodes with a high number of connections [123]. The network developed in our study demonstrates heterogeneity. Observed thickenings reflect the associated accumulation of amino acids, sterols, and carboxylates (cluster 1). Metabolically, these compounds are far from each other, but they turn out to be functionally related. Apparently, sterols and amino acids are actively involved in synthetic pathways at the initial stage and are the result of degradation at the final stages of cell development. Carboxylic acids are associated with both the synthesis and breakdown of amino acids and sterols. The association of sucrose, fructose, and glucose in one cluster (cluster 2) is not surprising. But the inclusion of FFAs in this cluster requires additional analysis. These metabolites are assumed to be involved in the processes of elongation growth (from initiation until completion). An important sign of this stage is the accumulation of sugars in intracellular compartments, the restructuring and metabolism of carbohydrates, including the synthesis and structural alteration in cell wall composition, as well as the dynamics of synthesis and reorganization of cell membranes. Cluster 3 unites metabolites which are characterized by an elevation in their content during the period of active elongation growth, and during further senescence and death. The presence of pyruvate, monosaccharides, and glycosides, as well as some carboxylates, should be noted. Such association of metabolites is probably related to the activation of sugar metabolism, including glycolysis. A similar pattern was determined for nitrogenous bases and putrescine.

The revealed common trends of metabolically unrelated compounds may reflect a global systemic factor. The phenomenon of a high level of correlation for such metabolites has been noted before [119,124]. It is associated with systemic rearrangements of various metabolic processes during development. Trophic and hormonal signaling pathways are assumed to be regulatory integration factors. The resulting network shows an interesting pattern: some biochemically related compounds are concentrated in the same or close clusters, as in the case of amino acids, sterols, and FFAs. A similar grouping of metabolites, according to chemical and metabolic specificity, has already been observed previously [125,126]. Alternatively, other, biochemically related compounds could be largely “dispersed” over the network. For example, pyruvate, citrate, and other TCA cycle intermediates show rather different patterns of dynamics. Glycosides are also scattered throughout the clusters. But still the important question arises: how is metabolic proximity associated with the revealed correlation of accumulation during development?

### 3.11. The Relationship Between the Chemical Nature of Metabolites and Correlations of Their Accumulation

To be able to answer this question, we compare the variability in metabolite content, its chemical specificity, and affiliation to metabolic pathways. An important observation is that correlations within metabolic groups are shifted in a positive direction (Figure 8, “inside”). Thus, the nature of the biochemical pathway contributes to the determination of correlations of the metabolite representation. The possibility of independent alteration in the metabolite accumulation is probably limited by metabolic fluxes, compartmentalization, and dependence on common resources. This supposition is in agreement with a connection between the network topology reconstructed by correlations and known biochemical pathways [127,128,129]. In our study, the distribution of correlations was different in various pathways (Figure 8). A high level of correlations was shown by a number of specialized pathways, for example, fatty acid and sterol metabolism. These pathways have a relatively small number of reactions and include biochemically related metabolites. The direction of metabolic fluxes into them quickly fills all the pools, although these paths are isolated from others. The case with amino acids is more complicated. It is noteworthy that the levels of these metabolites vary more or less consistently, despite the fact that their precursors are intermediates of the TCA cycle and glycolysis, which show less consistency in changes. Probably, the key importance shifts from the structure of the metabolic network to functional regulation associated with the synthesis and degradation of proteins. For some groups of metabolites, there was no increase in correlations, especially for central metabolic nodes such as pyruvate metabolism. Possibly, those metabolites are in the cross-interaction of several active pathways that differ in compartmentalization and complexity of regulation.

### 3.12. Patterns of Individual Variability Reflect the Functional State of Cells

Random variability in the content of metabolites between individual plants or cell cultures is rarely considered. According to the experimental data, the accumulation of metabolites in cell cultures, even under the same conditions and in the same phase of cell development, show diversity. Correlations in the metabolite accumulation detected in parallel subculturing reflected the patterns of enzymatic activity and flux intensity that developed according to the current conditions and previous cultivation. Thus, the pattern of correlations could be considered as a fingerprint characterizing the state of a biological system [130]. Those patterns have been shown to be specific to organs and tissues [125,130], to genotype [125,131], and to environmental conditions [127,129]. Subsequent assessment of the similarity of the accumulation patterns for pairs of metabolites by Canberra distance is visualized as heat maps (Figure 9). Significant changes in the patterns during the BY-2 culturing stages were determined. Cluster analysis at different time points based on pattern similarity revealed similarities previously observed when metabolite profiles were compared. The differences between the lag phase, proliferation, elongation growth, and aging are traced. Furthermore, the “death” phase exhibited distinct metabolic specificity. This indicates that development is associated with a cross-interaction between the pools of metabolites, which is constantly adjusted to varying internal and external factors. Thus, coordinated changes in the metabolite accumulation are an integral property of the biological system, since they unite all metabolic reactions and regulatory processes [119,121,124].

## 4. Materials and Methods

### 4.1. Plant Material

A heterotrophic cell suspension culture of *Nicotiana tabacum* L. (BY-2, Bright, Yellow; kindly gifted by Prof. E. Zazimalova). Cultures were maintained in Murashige and Skoog medium [1] supplemented with 30 g/L sucrose in 250 mL Erlenmeyer flasks containing 50 mL of medium. Cultures were kept in the dark, at a constant temperature of 26 °C and on a rotary shaker (120 rpm). Cells (5 mL) were inoculated in fresh medium every 3 weeks. For experiments, the initial fresh weight density was adjusted to 10–12 mg/L. Experimental cultures were maintained in 500 mL Erlenmeyer flasks with 110 mL of medium. Approximately 200 mg of fresh biomass was sampled by filtration. Samples were taken from cultures of 1, 2, 3, 4, 7, 14, 18, 21, 23, 26 days after inoculation. The experiment was conducted in five biological replicates. Samples were rapidly frozen in liquid nitrogen.

### 4.2. Metabolite Profiling

Cells were disrupted in a ball mill (Tissue Lyser LT, QIAGEN, Hilden, Germany). Extraction was performed with a chilled mixture of methanol–chloroform–water in the ratio of 5:2:2. After extraction, samples were cleared of cell debris by centrifugation for 10 min, 15,000× *g* at 4 °C. The extract was evaporated in a vacuum evaporator (Labconco, Kansas City, MO, US). The dried material was dissolved in a mixture of pyridine and silylating mix of BSFA:TMCS 99:1 (Sigma, St. Louis, MO, USA), with the addition of an internal standard (tricosan, normal C_23_ hydrocarbon, Sigma). The material was derivatized by incubating the samples at 90 °C for 20 min.

GC-MS analysis was performed with an Agilent 6850 gas chromatograph coupled to an Agilent 5975 mass spectrometer (Agilent Technologies, Santa Clara, CA, USA). Separation was performed on a Rxi-Sil-5ms capillary column (RESTEK, Bellefonte, PA, USA). The inlet temperature was −250 °C, in splitless mode; the column thermostat was set at an initial temperature of 70 °C, then a linear increase at a rate of 6°/min up to 320 °C.

Data were analyzed with the PARADISe 6.0.1 software [132] coupled with NIST MS Search 2.4 (National Institute of Standards and Technology, NIST, Santa Clara, CA, USA). For additional metabolite annotation, the AMDIS (Automated Mass Spectral Deconvolution and Identification System, NIST, Santa Clara, CA, USA) was used. Identification was made by mass spectra and RI match with records in libraries: NIST2020 [133], Golm Metabolome Database [134] and in-house library of the laboratory of analytical phytochemistry BIN RAS. Mass spectra were attributed to the compound if the match factor of the similarity to the library record was at least 800 (80 for AMDIS). If a mass spectrum was similar to several members of a class, it was annotated with class and RI (hexose_RI=, sterol_RI=, etc.). The unannotated compounds were used in the analysis as labeled by retention indices (“na_RI=”).

### 4.3. Monitoring Sucrose and Hexose Concentrations in the Medium 

The concentrations of sucrose, glucose, and fructose in the culture medium in samples for GC-MS were measured by HPLC-ELSD with a LicArt 62 (Labconcept, St. Petersburg, Russia), on an Inspire™ HILIC column (Dikma Technologies, Foothill Ranch, CA, USA). Mobile phase “A” was a mix of water/acetonitrile/formic acid 95:5:0.1; “B”—acetonitrile/water/formic acid 90:10:0.1. The gradient elution mode was 90% “B” (5 min); 80% (10 min); 10% (20 min); 10% (30 min); 90% (35 min); 90% (40 min), at a flow rate of 300 μL/min and column temperature of 30 °C. The injection volume was 5 μL. Detection was performed on an evaporative light scattering detector (ELSD), carrier gas (nitrogen) flow rate 3.0 L/min, and evaporation temperature 60 °C. Identification was performed by comparing the retention times of the peaks with the peaks of the standards. Quantification was made from peak areas calibrated with standards.

### 4.4. Data Analysis

The data were analyzed in the R 4.3.1 ‘Beagle Scouts’ environment. The data were normalized by the sample median, the logarithm taken, and standardized. Normalized peak areas are displayed in Supplementary Table S1. If a compound was absent in a sample but present in the other replicates, this was considered a technical error and impute was performed using the KNN (k-nearest neighbors) method using the impute package [135]. A principal component analysis (PCA) was performed using pcaMethods [136]. An OPLS-DA (orthogonal projections in latent structure—discriminant analysis) was performed using the ropls package [137]. The fgsea package [138] was used for a metabolite set enrichment analysis (MSEA). The metabolite sets for biochemical pathways for MSEA were downloaded from the KEGG database [139] using the KEGGREST package [140]. The list of metabolites belonging to different biochemical pathways was manually corrected, as the required pathways were added for some metabolites. Compounds for which a class was annotated were placed in the corresponding pathways. The metabolic map was built on the Cytoscape 3.10.2 software [141].

## 5. Conclusions

The cells of the BY-2 suspension culture experience complex systemic rearrangements, including changes in the metabolome. The lag phase, proliferation phase, elongation growth phase, stationary phase, and death are well manifested. One of the factors affecting metabolic rearrangements is a change in trophic status. Tobacco cells rapidly break down sucrose extracellularly and uptake sugars. The initial period of development is characterized by the accumulation of free amino acids and sterols, which is in agreement with the synthetic activity during the absorption of the substrate. The strongest changes occur after the completion of division and the activation of elongation growth, when sucrose in the medium is eliminated. Further active elongation mainly involves the activation of sugar metabolism and the repression of synthetic processes. The completion of elongation growth is accompanied by an increase in the level of metabolites associated with specialized metabolism and the formation of a secondary cell wall. In the stationary phase, there is a secondary increase in the accumulation of amino acids, sterols, and FFAs due to destructive processes. Correlations in the dynamics of the metabolite content during the process of culture growth and cell development are determined both by their functional connections and the structure of biochemical pathways. The obtained data are clearly related to cross-connections of metabolic pools and its dynamic alterations during the development of the BY-2 suspension culture.

## Data Availability

Data used for metabolomic analysis are available in Supplementary Materials, Table S1. Additional data will be made available by the authors on request.

## Supplementary Materials

The following supporting information can be downloaded at: https://www.mdpi.com/article/10.3390/plants13233426/s1, Table S1. Normalized peak areas. Table S2. OPLS-DA models. Table S3. Biomass density. Figure S1. Stigmasterol/β-sitosterol ratios.

## References

[1] Murashige T., Skoog F. (1962). A Revised Medium for Rapid Growth and Bio Assays with Tobacco Tissue Cultures. Physiol. Plant..

[2] Phillips G.C., Garda M. (2019). Plant Tissue Culture Media and Practices: An Overview. Vitro Cell. Dev. Biol.-Plant.

[3] Fehér A. (2019). Callus, Dedifferentiation, Totipotency, Somatic Embryogenesis: What These Terms Mean in the Era of Molecular Plant Biology?. Front. Plant Sci..

[4] Santos R.B., Abranches R., Fischer R., Sack M., Holland T. (2016). Putting the Spotlight Back on Plant Suspension Cultures. Front. Plant Sci..

[5] Malerba M., Cerana R. (2021). Plant Cell Cultures as a Tool to Study Programmed Cell Death. Int. J. Mol. Sci..

[6] Čuperlović-Culf M., Barnett D.A., Culf A.S., Chute I. (2010). Cell Culture Metabolomics: Applications and Future Directions. Drug Discov. Today.

[7] Meyer H., Schmidhalter D.R. (2014). Industrial Scale Suspension Culture of Living Cells.

[8] Wu T., Kerbler S.M., Fernie A.R., Zhang Y. (2021). Plant Cell Cultures as Heterologous Bio-Factories for Secondary Metabolite Production. Plant Commun..

[9] Malla A., Shanmugaraj B., Srinivasan B., Sharma A., Ramalingam S. (2020). Metabolic Engineering of Isoflavonoid Biosynthesis by Expressing Glycine Max Isoflavone Synthase in *Allium cepa* L. for Genistein Production. Plants.

[10] Häkkinen S.T., Reuter L., Nuorti N., Joensuu J.J., Rischer H., Ritala A. (2018). Tobacco BY-2 Media Component Optimization for a Cost-Efficient Recombinant Protein Production. Front. Plant Sci..

[11] Zagorskaya A.A., Deineko E.V. (2017). Suspension-Cultured Plant Cells as a Platform for Obtaining Recombinant Proteins. Russ. J. Plant Physiol..

[12] Häkkinen S.T., Nygren H., Nohynek L., Puupponen-Pimiä R., Heiniö R.-L., Maiorova N., Rischer H., Ritala A. (2020). Plant Cell Cultures as Food—Aspects of Sustainability and Safety. Plant Cell Rep..

[13] Gubser G., Vollenweider S., Eibl D., Eibl R. (2021). Food Ingredients and Food Made with Plant Cell and Tissue Cultures: State-of-the Art and Future Trends. Eng. Life Sci..

[14] Menges M., Murray J.A.H. (2006). Synchronization, Transformation, and Cryopreservation of Suspension-Cultured Cells. Arabidopsis Protocols.

[15] Hasnain A., Naqvi S.A.H., Ayesha S.I., Khalid F., Ellahi M., Iqbal S., Hassan M.Z., Abbas A., Adamski R., Markowska D. (2022). Plants in Vitro Propagation with Its Applications in Food, Pharmaceuticals and Cosmetic Industries; Current Scenario and Future Approaches. Front. Plant Sci..

[16] Nagata T., Nemoto Y., Hasezawa S. (1992). Tobacco BY-2 Cell Line as the “HeLa” Cell in the Cell Biology of Higher Plants. International Review of Cytology.

[17] Miyazawa Y., Sakai A., Nagata T., Matsuoka K., Inzé D. (2006). Tobacco BY-2 Cells as a Model for Differentiation in Heterotrophic Plant Cells. Tobacco BY-2 Cells: From Cellular Dynamics to Omics.

[18] Nagata T. (2023). Hidden History of the Tobacco BY-2 Cell Line. J. Plant Res..

[19] Srba M., Černíková A., Opatrný Z., Fischer L. (2016). Practical Guidelines for the Characterization of Tobacco BY-2 Cell Lines. Biol. Plant..

[20] Zažimalová E., Opatrný Z., Březinová A., Eder J. (1995). The Effect of Auxin Starvation on the Growth of Auxin-Dependent Tobacco Cell Culture: Dynamics of Auxin-Binding Activity and Endogenous Free IAA Content. J. Exp. Bot..

[21] Nagata T., Kumagai F. (1999). Plant Cell Biology through the Window of the Highly Synchronized Tobacco BY-2 Cell Line. Methods Cell Sci..

[22] Tsuchiya Y., Nakamura T., Izumi Y., Okazaki K., Shinano T., Kubo Y., Katsuhara M., Sasaki T., Yamamoto Y. (2021). Physiological Role of Aerobic Fermentation Constitutively Expressed in an Aluminum-Tolerant Cell Line of Tobacco (*Nicotiana tabacum*). Plant Cell Physiol..

[23] Toyooka K., Sato M., Wakazaki M., Matsuoka K. (2016). Morphological and Quantitative Changes in Mitochondria, Plastids, and Peroxisomes during the Log-to-Stationary Transition of the Growth Phase in Cultured Tobacco BY-2 Cells. Plant Signal. Behav..

[24] Suzuki T., Kawano S., Sakai A., Fujie M., Kuroiwa H., Nakamura H., Kuroiwa T. (1992). Preferential Mitochondrial and Plastid Dna Synthesis before Multiple Cell Divisions in *Nicotiana tabacum*. J. Cell Sci..

[25] Matsuoka K., Demura T., Galis I., Horiguchi T., Sasaki M., Tashiro G., Fukuda H. (2004). A Comprehensive Gene Expression Analysis Toward the Understanding of Growth and Differentiation of Tobacco BY-2 Cells. Plant Cell Physiol..

[26] Puzanskiy R.K., Kirpichnikova A.A., Shavarda A.L., Yemelyanov V.V., Shishova M.F. (2024). Senescence Metabolomics of *Nicotiana tabacum* L. VBI-0 Heterotrophic Suspension Cultures. Ecol. Gen..

[27] Gemperlová L., Eder J., Cvikrová M. (2005). Polyamine Metabolism during the Growth Cycle of Tobacco BY-2 Cells. Plant Physiol. Biochem..

[28] Hensel G., Kunze G., Kunze I. (2002). The Influence of 2,4-Dichlorophenoxyacetic Acid on Localisation of the PR-Proteins CBP20 and Class I Chitinase in Tobacco Suspension Cell Cultures. Plant Sci..

[29] Issawi M., Muhieddine M., Girard C., Sol V., Riou C. (2017). Unexpected Features of Exponentially Growing Tobacco Bright Yellow-2 Cell Suspension Culture in Relation to Excreted Extracellular Polysaccharides and Cell Wall Composition. Glycoconj. J..

[30] Puzanskiy R.K., Romanyuk D.A., Kirpichnikova A.A., Yemelyanov V.V., Shishova M.F. (2024). Plant Heterotrophic Cultures: No Food, No Growth. Plants.

[31] Contento A.L., Kim S.-J., Bassham D.C. (2004). Transcriptome Profiling of the Response of Arabidopsis Suspension Culture Cells to Suc Starvation. Plant Physiol..

[32] Journet E.P., Bligny R., Douce R. (1986). Biochemical Changes during Sucrose Deprivation in Higher Plant Cells. J. Biol. Chem..

[33] Gout E., Bligny R., Douce R., Boisson A., Rivasseau C. (2011). Early Response of Plant Cell to Carbon Deprivation: In Vivo^31^ P-NMR Spectroscopy Shows a Quasi-instantaneous Disruption on Cytosolic Sugars, Phosphorylated Intermediates of Energy Metabolism, Phosphate Partitioning, and Intracellular pHs. New Phytol..

[34] Wang H.-J., Wan A.-R., Hsu C.-M., Lee K.-W., Yu S.-M., Jauh G.-Y. (2007). Transcriptomic Adaptations in Rice Suspension Cells under Sucrose Starvation. Plant Mol. Biol..

[35] Kim S.W., Koo B.C., Kim J., Liu J.R. (2007). Metabolic Discrimination of Sucrose Starvation from *Arabidopsis* Cell Suspension by1H NMR Based Metabolomics. Biotechnol. Bioprocess Eng..

[36] Binder S. (2010). Branched-Chain Amino Acid Metabolism in *Arabidopsis thaliana*. Arab. Book.

[37] Morkunas I., Borek S., Formela M., Ratajczak L., Chang C.-F. (2012). Plant Responses to Sugar Starvation. Carbohydrates—Comprehensive Studies on Glycobiology and Glycotechnology.

[38] Fiehn O. (2016). Metabolomics by Gas Chromatography–Mass Spectrometry: Combined Targeted and Untargeted Profiling. CP Mol. Biol..

[39] De Gunst M.C.M., Harkes P.A.A., Val J., Van Zwet W.R., Libbenga K.R. (1990). Modelling the Growth of a Batch Culture of Plant Cells: A Corpuscular Approach. Enzyme Microb. Technol..

[40] Shibasaki N., Obika R., Yonemoto T., Tadaki T. (1995). Kinetic Analysis for Effect of Initial Substrate Concentration on Growth and Secondary Metabolite Production in Cultures of *Nicotiana tabacum*. J. Chem. Technol. Biotechnol..

[41] Wink M. (1994). The Cell Culture Medium? A Functional Extracellular Compartment of Suspension-Cultured Cells. Plant Cell Tissue Organ Cult..

[42] Hasezawa S., Syōno K. (1983). Hormonal Control of Elongation of Tobacco Cells Derived from Protoplasts. Plant Cell Physiol..

[43] Miyazawa Y., Sakai A., Miyagishima S., Takano H., Kawano S., Kuroiwa T. (1999). Auxin and Cytokinin Have Opposite Effects on Amyloplast Development and the Expression of Starch Synthesis Genes in Cultured Bright Yellow-2 Tobacco Cells. Plant Physiol..

[44] Miyazawa Y., Kutsuna N., Inada N., Kuroiwa H., Kuroiwa T., Yoshida S. (2002). Dedifferentiation of Starch-Storing Cultured Tobacco Cells: Effects of 2,4-Dichlorophenoxy Acetic Acid on Multiplication, Starch Content, Organellar DNA Content, and Starch Synthesis Gene Expression. Plant Cell Rep..

[45] Sakai A., Yashiro K., Kawano S., Kuroiwa T. (1996). Amyloplast Formation in Cultured Tobacco Cells; Effects of Plant Hormones on Multiplication, Size, and Starch Content. Plant Cell Rep..

[46] Fiehn O. (2002). Metabolomics—The link between genotypes and phenotypes. Plant Mol. Biol..

[47] Weckwerth W. (2010). Metabolomics: An Integral Technique in Systems Biology. Bioanalysis.

[48] Salam U., Ullah S., Tang Z.-H., Elateeq A.A., Khan Y., Khan J., Khan A., Ali S. (2023). Plant Metabolomics: An Overview of the Role of Primary and Secondary Metabolites against Different Environmental Stress Factors. Life.

[49] Yurkov A.P., Puzanskiy R.K., Avdeeva G.S., Jacobi L.M., Gorbunova A.O., Kryukov A.A., Kozhemyakov A.P., Laktionov Y.V., Kosulnikov Y.V., Romanyuk D.A. (2021). Mycorrhiza-Induced Alterations in Metabolome of Medicago Lupulina Leaves during Symbiosis Development. Plants.

[50] Petrova N.V., Sazanova K.V., Medvedeva N.A., Shavarda A.L. (2019). Features of Metabolomic Profiles in Different Stages of Ontogenesis in *Prunella vulgaris* (Lamiaceae) Grown in a Climate Chamber. Russ. J. Bioorgan. Chem..

[51] Liu A., Yuan K., Li Q., Liu S., Li Y., Tao M., Xu H., Tian J., Guan S., Zhu W. (2022). Metabolomics and Proteomics Revealed the Synthesis Difference of Aroma Precursors in Tobacco Leaves at Various Growth Stages. Plant Physiol. Biochem..

[52] Li L., Zhao J., Zhao Y., Lu X., Zhou Z., Zhao C., Xu G. (2016). Comprehensive Investigation of Tobacco Leaves during Natural Early Senescence via Multi-Platform Metabolomics Analyses. Sci. Rep..

[53] Li W., Zhang H., Li X., Zhang F., Liu C., Du Y., Gao X., Zhang Z., Zhang X., Hou Z. (2017). Intergrative Metabolomic and Transcriptomic Analyses Unveil Nutrient Remobilization Events in Leaf Senescence of Tobacco. Sci. Rep..

[54] Zhang L., Zhang X., Ji H., Wang W., Liu J., Wang F., Xie F., Yu Y., Qin Y., Wang X. (2018). Metabolic Profiling of Tobacco Leaves at Different Growth Stages or Different Stalk Positions by Gas Chromatography–Mass Spectrometry. Ind. Crops Prod..

[55] Hao J., Wang X., Chai Y., Huang X., Wu H., Zhang S., Duan X., Qin L. (2024). Evaluation of Lipid and Metabolite Profiles in Tobacco Leaves from Different Plant Parts by Comprehensive Lipidomics and Metabolomics Analysis. Ind. Crops Prod..

[56] Naoumkina M., Hinchliffe D.J., Turley R.B., Bland J.M., Fang D.D. (2013). Integrated Metabolomics and Genomics Analysis Provides New Insights into the Fiber Elongation Process in Ligon Lintless-2 Mutant Cotton (*Gossypium hirsutum* L.). BMC Genom..

[57] Lalonde S., Wipf D., Frommer W.B. (2004). Transport mechanisms for organic forms of carbon and nitrogen between source and sink. Annu. Rev. Plant Biol..

[58] Dominguez P.G., Niittylä T. (2022). Mobile Forms of Carbon in Trees: Metabolism and Transport. Tree Physiol..

[59] Lamport D.T.A. (1964). Cell Suspension Cultures of Higher Plants: Isolation and Growth Energetics. Exp. Cell Res..

[60] Koiwai A., Tanno Y., Noguchi M. (1970). The Effects of Amino Acids on the Growth and Glutamic Acid Decarboxylase Activity of Tobacco Cell Cultures. Agric. Biol. Chem..

[61] Schenk R.U., Hildebrandt A.C. (1972). Medium and Techniques for Induction and Growth of Monocotyledonous and Dicotyledonous Plant Cell Cultures. Can. J. Bot..

[62] Milne R.J., Grof C.P., Patrick J.W. (2018). Mechanisms of Phloem Unloading: Shaped by Cellular Pathways, Their Conductances and Sink Function. Curr. Opin. Plant Biol..

[63] Ma S., Li Y., Li X., Sui X., Zhang Z. (2019). Phloem Unloading Strategies and Mechanisms in Crop Fruits. J. Plant Growth Regul..

[64] Ji J., Yang L., Fang Z., Zhang Y., Zhuang M., Lv H., Wang Y. (2022). Plant SWEET Family of Sugar Transporters: Structure, Evolution and Biological Functions. Biomolecules.

[65] Bavnhøj L., Driller J.H., Zuzic L., Stange A.D., Schiøtt B., Pedersen B.P. (2023). Structure and Sucrose Binding Mechanism of the Plant SUC1 Sucrose Transporter. Nat. Plants.

[66] Yamanaka A., Hashimoto A., Matsuo T., Kanou M., Suehara K.-I., Kameoka T. (2007). Analysis of Kinetic Uptake Phenomena of Monosaccharide and Disaccharide by Suspension TBY-2 Cells Using an FT-IR/ATR Method. Bioprocess Biosyst. Eng..

[67] Katō K., Noguchi M. (1976). Sugar Composition of Cell Wall Polysaccharides of Suspension-Cultured Tobacco Cells. Agric. Biol. Chem..

[68] Mohamad Puad N.I., Abd-Karim K., Mavituna F. (2017). A Model for Arabidopsis Thaliana Cell Suspension Growth and Sugar Uptake Kinetics. J. Teknol..

[69] Arias J.P., Mendoza D., Arias M. (2021). Agitation Effect on Growth and Metabolic Behavior of Plant Cell Suspension Cultures of Thevetia Peruviana at Bench Scale Reactor. Plant Cell Tissue Organ Cult..

[70] Madhusudhan R., Ramachandra Rao S., Ravishankar G.A. (1995). Osmolarity as a Measure of Growth of Plant Cells in Suspension Cultures. Enzyme Microb. Technol..

[71] Bertrand R.L. (2019). Lag Phase Is a Dynamic, Organized, Adaptive, and Evolvable Period That Prepares Bacteria for Cell Division. J. Bacteriol..

[72] Muselikova K., Mouralova K. (2024). Synthetic Auxin Herbicide 2,4-D and Its Influence on a Model BY-2 Suspension. Mol. Biol. Rep..

[73] Oesterhelt C., Schnarrenberger C., Gross W. (1999). Characterization of a Sugar/Polyol Uptake System in the Red Alga *Galdieria sulphuraria*. Eur. J. Phycol..

[74] Tanner W., Sauer N. (1989). [26] Uptake of Sugars and Amino Acids by Chlorella. Methods in Enzymology.

[75] Gibson S.I. (2003). Sugar and Phytohormone Response Pathways: Navigating a Signalling Network. J. Exp. Bot..

[76] Sakr S., Wang M., Dédaldéchamp F., Perez-Garcia M.-D., Ogé L., Hamama L., Atanassova R. (2018). The Sugar-Signaling Hub: Overview of Regulators and Interaction with the Hormonal and Metabolic Network. Int. J. Mol. Sci..

[77] Creydt M., Fischer M. (2020). Metabolic Imaging: Analysis of Different Sections of White Asparagus Officinalis Shoots Using High-Resolution Mass Spectrometry. J. Plant Physiol..

[78] Momoyama Y., Miyazawa Y., Miyagishima S., Mori T., Misumi O., Kuroiwa H., Kuroiwa T. (2003). The Division of Pleomorphic Plastids with Multiple FtsZ Rings in Tobacco BY-2 Cells. Eur. J. Cell Biol..

[79] Satoh M., Nemoto Y., Kawano S., Nagata T., Hirokawa H., Kuroiwa T. (1993). Organization of Heterogeneous Mitochondrial DNA Molecules in Mitochondrial Nuclei of Cultured Tobacco Cells. Protoplasma.

[80] Rontein D., Dieuaide-Noubhani M., Dufourc E.J., Raymond P., Rolin D. (2002). The Metabolic Architecture of Plant Cells. J. Biol. Chem..

[81] Colombié S., Nazaret C., Bénard C., Biais B., Mengin V., Solé M., Fouillen L., Dieuaide-Noubhani M., Mazat J., Beauvoit B. (2015). Modelling Central Metabolic Fluxes by Constraint-based Optimization Reveals Metabolic Reprogramming of Developing *Solanum lycopersicum* (Tomato) Fruit. Plant J..

[82] Shameer S., Vallarino J.G., Fernie A.R., Ratcliffe R.G., Sweetlove L.J. (2020). Flux Balance Analysis of Metabolism during Growth by Osmotic Cell Expansion and Its Application to Tomato Fruits. Plant J..

[83] Puzanskiy R., Tarakhovskaya E., Shavarda A., Shishova M. (2018). Metabolomic and Physiological Changes of Chlamydomonas Reinhardtii (Chlorophyceae, Chlorophyta) during Batch Culture Development. J. Appl. Phycol..

[84] Shtark O.Y., Puzanskiy R.K., Avdeeva G.S., Yurkov A.P., Smolikova G.N., Yemelyanov V.V., Kliukova M.S., Shavarda A.L., Kirpichnikova A.A., Zhernakov A.I. (2019). Metabolic Alterations in Pea Leaves during Arbuscular Mycorrhiza Development. PeerJ.

[85] Liu Y., Li M., Xu J., Liu X., Wang S., Shi L. (2019). Physiological and Metabolomics Analyses of Young and Old Leaves from Wild and Cultivated Soybean Seedlings under Low-Nitrogen Conditions. BMC Plant Biol..

[86] Igamberdiev A.U., Bykova N.V. (2018). Role of Organic Acids in the Integration of Cellular Redox Metabolism and Mediation of Redox Signalling in Photosynthetic Tissues of Higher Plants. Free Radic. Biol. Med..

[87] Igamberdiev A.U., Eprintsev A.T. (2016). Organic Acids: The Pools of Fixed Carbon Involved in Redox Regulation and Energy Balance in Higher Plants. Front. Plant Sci..

[88] Zhang Y., Fernie A.R. (2023). The Role of TCA Cycle Enzymes in Plants. Adv. Biol..

[89] Fragallah S.A.D.A., Wang P., Li N., Chen Y., Lin S. (2018). Metabolomic Analysis of Pollen Grains with Different Germination Abilities from Two Clones of Chinese Fir (*Cunninghamia lanceolata* (Lamb) Hook). Molecules.

[90] Du Y., Fu X., Chu Y., Wu P., Liu Y., Ma L., Tian H., Zhu B. (2022). Biosynthesis and the Roles of Plant Sterols in Development and Stress Responses. Int. J. Mol. Sci..

[91] Hartmann M. (1998). Plant Sterols and the Membrane Environment. Trends Plant Sci..

[92] Mongrand S., Morel J., Laroche J., Claverol S., Carde J.-P., Hartmann M.-A., Bonneu M., Simon-Plas F., Lessire R., Bessoule J.-J. (2004). Lipid Rafts in Higher Plant Cells. J. Biol. Chem..

[93] Aboobucker S.I., Suza W.P. (2019). Why Do Plants Convert Sitosterol to Stigmasterol?. Front. Plant Sci..

[94] Griebel T., Zeier J. (2010). A Role for Β-sitosterol to Stigmasterol Conversion in Plant–Pathogen Interactions. Plant J..

[95] Gamalero E., Lingua G., Glick B.R. (2023). Ethylene, ACC, and the Plant Growth-Promoting Enzyme ACC Deaminase. Biology.

[96] Vanderstraeten L., Depaepe T., Bertrand S., Van Der Straeten D. (2019). The Ethylene Precursor ACC Affects Early Vegetative Development Independently of Ethylene Signaling. Front. Plant Sci..

[97] Van De Poel B., Van Der Straeten D. (2014). 1-Aminocyclopropane-1-Carboxylic Acid (ACC) in Plants: More than Just the Precursor of Ethylene!. Front. Plant Sci..

[98] Fomenkov A.A., Nosov A.V., Rakitin V.Y., Sukhanova E.S., Mamaeva A.S., Sobol’kova G.I., Nosov A.M., Novikova G.V. (2015). Ethylene in the Proliferation of Cultured Plant Cells: Regulating or Just Going Along?. Russ. J. Plant Physiol..

[99] Dubois M., Van Den Broeck L., Inzé D. (2018). The Pivotal Role of Ethylene in Plant Growth. Trends Plant Sci..

[100] Gamborg O.L., Larue T.A.G. (1971). Ethylene Production by Plant Cell Cultures: The Effect of Auxins, Abscisic Acid, and Kinetin on Ethylene Production in Suspension Cultures of Rose and *Ruta* Cells. Plant Physiol..

[101] Beauvoit B.P., Colombié S., Monier A., Andrieu M.-H., Biais B., Bénard C., Chéniclet C., Dieuaide-Noubhani M., Nazaret C., Mazat J.-P. (2014). Model-Assisted Analysis of Sugar Metabolism throughout Tomato Fruit Development Reveals Enzyme and Carrier Properties in Relation to Vacuole Expansion. Plant Cell.

[102] Biais B., Bénard C., Beauvoit B., Colombié S., Prodhomme D., Ménard G., Bernillon S., Gehl B., Gautier H., Ballias P. (2014). Remarkable Reproducibility of Enzyme Activity Profiles in Tomato Fruits Grown under Contrasting Environments Provides a Roadmap for Studies of Fruit Metabolism. Plant Physiol..

[103] Muñoz-Bertomeu J., Cascales-Miñana B., Mulet J.M., Baroja-Fernández E., Pozueta-Romero J., Kuhn J.M., Segura J., Ros R. (2009). Plastidial Glyceraldehyde-3-Phosphate Dehydrogenase Deficiency Leads to Altered Root Development and Affects the Sugar and Amino Acid Balance in Arabidopsis. Plant Physiol..

[104] Fleming A. (2006). Metabolic Aspects of Organogenesis in the Shoot Apical Meristem. J. Exp. Bot..

[105] Dong N., Lin H. (2021). Contribution of Phenylpropanoid Metabolism to Plant Development and Plant–Environment Interactions. J. Integr. Plant Biol..

[106] Peč J., Flores-Sanchez I.J., Choi Y.H., Verpoorte R. (2010). Metabolic Analysis of Elicited Cell Suspension Cultures of *Cannabis sativa* L. by 1H-NMR Spectroscopy. Biotechnol. Lett..

[107] Yemelyanov V.V., Puzanskiy R.K., Burlakovskiy M.S., Lutova L.A., Shishova M.F., Zhan X. (2021). Metabolic Profiling of Transgenic Tobacco Plants Synthesizing Bovine Interferon-Gamma. Metabolomics—Methodology and Applications in Medical Sciences and Life Sciences.

[108] Gorshkova T.A., Gurjanov O.P., Mikshina P.V., Ibragimova N.N., Mokshina N.E., Salnikov V.V., Ageeva M.V., Amenitskii S.I., Chernova T.E., Chemikosova S.B. (2010). Specific Type of Secondary Cell Wall Formed by Plant Fibers. Russ. J. Plant Physiol..

[109] Cosgrove D.J. (2024). Structure and Growth of Plant Cell Walls. Nat. Rev. Mol. Cell Biol..

[110] Toyooka K., Sato M., Kutsuna N., Higaki T., Sawaki F., Wakazaki M., Goto Y., Hasezawa S., Nagata N., Matsuoka K. (2014). Wide-Range High-Resolution Transmission Electron Microscopy Reveals Morphological and Distributional Changes of Endomembrane Compartments during Log to Stationary Transition of Growth Phase in Tobacco BY-2 Cells. Plant Cell Physiol..

[111] McLoughlin F., Marshall R.S., Ding X., Chatt E.C., Kirkpatrick L.D., Augustine R.C., Li F., Otegui M.S., Vierstra R.D. (2020). Autophagy Plays Prominent Roles in Amino Acid, Nucleotide, and Carbohydrate Metabolism during Fixed-Carbon Starvation in Maize. Plant Cell.

[112] Wang P., Wang T., Han J., Li M., Zhao Y., Su T., Ma C. (2021). Plant Autophagy: An Intricate Process Controlled by Various Signaling Pathways. Front. Plant Sci..

[113] Chen H., Dong J., Wang T. (2021). Autophagy in Plant Abiotic Stress Management. Int. J. Mol. Sci..

[114] Avin-Wittenberg T., Bajdzienko K., Wittenberg G., Alseekh S., Tohge T., Bock R., Giavalisco P., Fernie A.R. (2015). Global Analysis of the Role of Autophagy in Cellular Metabolism and Energy Homeostasis in Arabidopsis Seedlings under Carbon Starvation. Plant Cell.

[115] Havé M., Marmagne A., Chardon F., Masclaux-Daubresse C. (2016). Nitrogen Remobilisation during Leaf Senescence: Lessons from Arabidopsis to Crops. J. Exp. Bot..

[116] Finkemeier I., Sweetlove L.J. (2009). The Role of Malate in Plant Homeostasis. F1000 Biol. Rep..

[117] Voitsekhovskaja O.V., Schiermeyer A., Reumann S. (2014). Plant Peroxisomes Are Degraded by Starvation-Induced and Constitutive Autophagy in Tobacco BY-2 Suspension-Cultured Cells. Front. Plant Sci..

[118] Damoo D.Y., Durnford D.G. (2021). Long-Term Survival of Chlamydomonas Reinhardtii during Conditional Senescence. Arch. Microbiol..

[119] Rosato A., Tenori L., Cascante M., De Atauri Carulla P.R., Martins Dos Santos V.A.P., Saccenti E. (2018). From Correlation to Causation: Analysis of Metabolomics Data Using Systems Biology Approaches. Metabolomics.

[120] Kose F., Weckwerth W., Linke T., Fiehn O. (2001). Visualizing Plant Metabolomic Correlation Networks Using Clique–Metabolite Matrices. Bioinformatics.

[121] Camacho D., De La Fuente A., Mendes P. (2005). The Origin of Correlations in Metabolomics Data. Metabolomics.

[122] Barupal D.K., Haldiya P.K., Wohlgemuth G., Kind T., Kothari S.L., Pinkerton K.E., Fiehn O. (2012). MetaMapp: Mapping and Visualizing Metabolomic Data by Integrating Information from Biochemical Pathways and Chemical and Mass Spectral Similarity. BMC Bioinf..

[123] Jeong H., Tombor B., Albert R., Oltvai Z.N., Barabási A.-L. (2000). The Large-Scale Organization of Metabolic Networks. Nature.

[124] Steuer R. (2006). Review: On the Analysis and Interpretation of Correlations in Metabolomic Data. Briefings Bioinf..

[125] Fukushima A., Kusano M., Redestig H., Arita M., Saito K. (2011). Metabolomic Correlation-Network Modules in Arabidopsis Based on a Graph-Clustering Approach. BMC Syst. Biol..

[126] Chouhan N., Marriboina S., Kumari A., Singh P., Yadav R.M., Gupta K.J., Subramanyam R. (2023). Metabolomic Response to High Light from Pgrl1 and Pgr5 Mutants of Chlamydomonas Reinhardtii. Photochem. Photobiol. Sci..

[127] Szymanski J., Jozefczuk S., Nikoloski Z., Selbig J., Nikiforova V., Catchpole G., Willmitzer L. (2009). Stability of Metabolic Correlations under Changing Environmental Conditions in *Escherichia coli*—A Systems Approach. PLoS ONE.

[128] Yang H.-F., Zhang X.-N., Li Y., Zhang Y.-H., Xu Q., Wei D.-Q. (2017). Theoretical Studies of Intracellular Concentration of Micro-Organisms’ Metabolites. Sci. Rep..

[129] Kotze H.L., Armitage E.G., Sharkey K.J., Allwood J.W., Dunn W.B., Williams K.J., Goodacre R. (2013). A Novel Untargeted Metabolomics Correlation-Based Network Analysis Incorporating Human Metabolic Reconstructions. BMC Syst. Biol..

[130] Morgenthal K., Weckwerth W., Steuer R. (2006). Metabolomic Networks in Plants: Transitions from Pattern Recognition to Biological Interpretation. Biosystems.

[131] Weckwerth W., Wenzel K., Fiehn O. (2004). Process for the Integrated Extraction, Identification and Quantification of Metabolites, Proteins and RNA to Reveal Their Co-regulation in Biochemical Networks. Proteomics.

[132] Johnsen L.G., Skou P.B., Khakimov B., Bro R. (2017). Gas Chromatography—Mass Spectrometry Data Processing Made Easy. J. Chromatogr. A.

[133] Evans H., Greene K.K., Healy W.M., Hoffman E., Rimmer K., Sberegaeva A., Zimmerman N.M. (2021). National Institute of Standards and Technology Environmental Scan 2020.

[134] Hummel J., Selbig J., Walther D., Kopka J., Nielsen J., Jewett M.C. (2007). The Golm Metabolome Database: A Database for GC-MS Based Metabolite Profiling. Metabolomics.

[135] Hastie T., Tibshirani R., Narasimhan B., Chu G. (2023). Impute: Imputation for Microarray Data, R Package Version 1.74.1.

[136] Stacklies W., Redestig H., Scholz M., Walther D., Selbig J. (2007). pcaMethods—A Bioconductor Package Providing PCA Methods for Incomplete Data. Bioinformatics.

[137] Thévenot E.A., Roux A., Xu Y., Ezan E., Junot C. (2015). Analysis of the Human Adult Urinary Metabolome Variations with Age, Body Mass Index, and Gender by Implementing a Comprehensive Workflow for Univariate and OPLS Statistical Analyses. J. Proteome Res..

[138] Korotkevich G., Sukhov V., Budin N., Shpak B., Artyomov M.N., Sergushichev A. (2016). Fast Gene Set Enrichment Analysis. bioRxiv.

[139] Kanehisa M., Furumichi M., Sato Y., Kawashima M., Ishiguro-Watanabe M. (2023). KEGG for Taxonomy-Based Analysis of Pathways and Genomes. Nucleic Acids Res..

[140] Tenenbaum D. (2017). KEGGREST: Client-Side REST Access to the Kyoto Encyclopedia of Genes and Genomes (KEGG), Version 1.46.0.

[141] Shannon P., Markiel A., Ozier O., Baliga N.S., Wang J.T., Ramage D., Amin N., Schwikowski B., Ideker T. (2003). Cytoscape: A Software Environment for Integrated Models of Biomolecular Interaction Networks. Genome Res..

