# Supplementary figures and images for: From Division to Death: Metabolomic Analysis of Nicotiana tabacum BY-2 Cells Reveals the Complexity of Life in Batch Culture
Source: Plants (Basel). 2024 Dec 6;13(23):3426. doi: 10.3390/plants13233426 (PMC11644078; doi:10.3390/plants13233426)

Figure S1. Stigmasterol/b-sitosterol ratios.

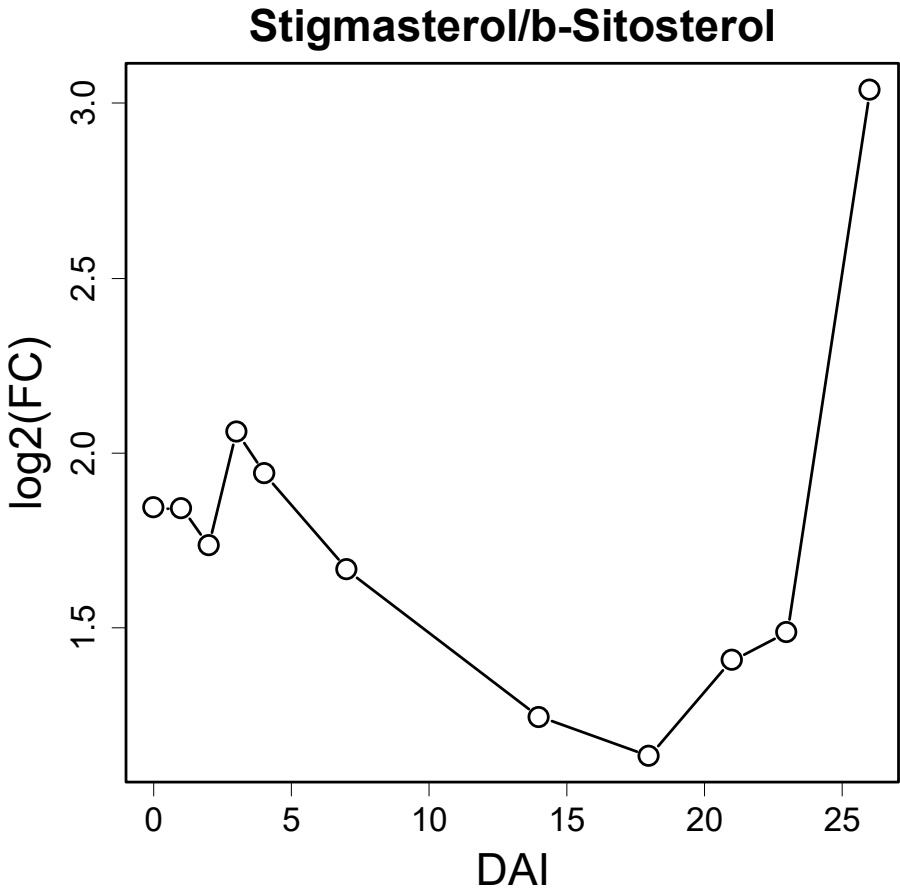

Supplement: Supplementary file 1 [file plants-13-03426-s001.zip › Figure S1.pdf]
